# An Integrative Analysis of the Role of Mitophagy in Comorbid Myocardial Infarction and Type 2 Diabetes Mellitus

**DOI:** 10.2174/0118715303431252260315125017

**Published:** 2025-04-08

**Authors:** Ying Yang, Feiyan Wan, Xuelian Xu, Wei Xu, Lingli Jiang, Pei Pan

**Affiliations:** 1 Department of Cardiology, Chinese Academy of Medical Sciences and Peking Union Medical College, Fuwai Hospital, Beijing, 100037, China;; 2 Department of Neurology, Guangzhou Eighth People's Hospital, Guangzhou Medical University No. 8, Huaying Road, Jiahe Street, Baiyun District, Guangzhou City, Guangdong Province, 510440, China;; 3 Department of Special Intensive Care Unit, The First Branch of the First Affiliated Hospital of Chongqing Medical University, Chongqing, 400042, China;; 4 Department of Cardiology, University-Town Hospital of Chongqing Medical University, Chongqing, 401331, China

**Keywords:** Myocardial infarction, type 2 diabetes mellitus, mitophagy, molecular mechanism, immune infiltration, cardiovascular disease

## Abstract

**Introduction:**

Coexisting myocardial infarction (MI) and type 2 diabetes mellitus (T2DM) is a common condition. We aimed to investigate the association between MI and type 2 diabetes (T2D) with mitophagy-related differentially expressed genes (MRDEGs) and the impact thereof on disease progression.

**Methods:**

R package was used for data retrieval and normalization of the MI (GSE48060 and GSE29532) and T2D (GSE76894 and GSE25724) datasets. Gene set enrichment analysis indicated significant associations between biological functions and pathways. Protein-protein interaction networks constructed using STRING and GeneMANIA revealed interconnected MRDEGs.

**Results:**

Principal component and differential gene expression analyses identified 45 MRDEGs. Through key gene screening, least absolute shrinkage, and selection operator (LASSO) risk model construction, nine MRDEGs were identified: *ATG14, BIRC2, CSK, FBXO7, HNRNPH1, HNRNPL, SNX6, TFEB,* and *YES1*. A diagnostic nomogram model exhibited high reliability and validity when utilizing these genes. Using LASSO regression analysis, the risk score formula demonstrated diagnostic accuracy for MI and T2D.

**Discussion:**

This study highlights the significance of immune cell infiltration in the context of MI and T2D. This analysis is primarily based on publicly available datasets, and future research will incorporate independent patient cohorts to assess the role of mitochondrial autophagy in these diseases.

**Conclusion:**

The screened genes may be therapeutic targets to prevent progression and improve clinical outcomes in patients with MI and T2D. This study provides an integrative view of mitochondrial metabolism and immune infiltration in MI and T2D, offering a novel direction to further elucidate the pathogenesis and medical interventions for these diseases.

## 1 INTRODUCTION

Cardiovascular disease (CVD) is the leading cause of disease burden worldwide, and the number of CVD deaths steadily reached 18.6 million in 2019 [1]. The American Heart Association estimates that an American person experiences a myocardial infarction (MI) approximately every 40 seconds [2].

More than 20 years ago, Jacoby *et al.* revealed a notable association between diabetes mellitus and coronary vascular disease, highlighting significant differences in the clinical progression of acute MI among diabetic patients compared to those without diabetes [3]. The cardiovascular mortality rate is more than twice as high in patients with diabetes as in those without, and MI accounts for approximately 30% of all deaths in patients with diabetes [3]. Hyperglycemia correlates with MI inflammatory indicators and infarct size [4]. Type 2 diabetes (T2D) is a common chronic disease. The International Diabetes Federation estimated that approximately 463 million adults had diabetes in 2019, and projections indicate that this number could reach 700 million by 2045 [5].

Coexisting MI and T2D are common in clinical settings. Many patients with CVD have undiagnosed T2DM. Given that having diabetes and CVD, especially at a younger age, has a major impact on prognosis, it is of utmost importance to screen patients with CVD for diabetes and to assess CV risk in individuals with diabetes [6, 7]. Similarly, patients with diabetes and insulin resistance are at a greater risk of developing CVDs [8]. A prospective follow-up study found that the risk of MI in patients with early onset T2D was 14- fold higher than that in controls [9]. Hyperglycaemia following ACS is associated with worse clinical outcomes [10]. The treatment of these diseases relies primarily on medications tailored to their respective pathogenic mechanisms. Therefore, it is imperative to identify potential common underlying mechanisms.

Autophagy is a crucial catabolic pathway in mammalian cells that is responsible for the breakdown and recycling of macromolecules and organelles, playing a vital role in eliminating protein aggregates and damaged or surplus organelles to maintain intracellular homeostasis and cellular health. However, autophagy occurs at minimal levels in the heart, and disruptions in this process can lead to cardiac dysfunction and heart failure [11]. The potential effect of mitochondrial autophagy or mitophagy on MI has gained attention, suggesting that dysregulation of this process may exacerbate injury and impair cardiac function [11-13].

In addition to its role in MI, mitophagy is crucial in other metabolic diseases such as T2D. Diabetes mellitus is not only a major risk factor for cardiovascular disease, but its complex metabolic abnormalities also promote the initiation and progression of coronary artery lesions through multiple mechanisms. In patients with type 2 diabetes, insulin resistance leads to vascular endothelial dysfunction, characterized by reduced nitric oxide production and impaired vasodilation, thereby accelerating arterial intimal injury and atherosclerosis. Persistent hyperglycemia activates the NF-κB signaling pathway, inducing the release of pro-inflammatory cytokines such as IL-6 and TNF-α, which creates a chronic low-grade inflammatory environment that exacerbates vascular wall inflammation and lipid deposition [14, 15]. Concurrently, the common dyslipidemia pattern in T2D—characterized by hypertriglyceridemia and low HDL-C levels—promotes the oxidation of low-density lipoprotein. Enhanced oxidative stress leads to reactive oxygen species accumulation, causing further damage to cardiomyocytes and vascular endothelium [16]. Additionally, increased platelet activation, elevated fibrinogen levels, and coagulation abnormalities in diabetes collectively promote thrombus formation, significantly increasing the risk of myocardial infarction. Microvascular complications and myocardial remodeling also serve as critical pathological bases for myocardial vulnerability in T2D patients, resulting in heterogeneous myocardial perfusion and reduced ischemic threshold [17]. These interconnected metabolic and vascular abnormalities collectively explain the significantly elevated risk of coronary artery disease and myocardial infarction in T2D patients, while also suggesting that dysregulated mitophagy may represent an underlying common molecular mechanism. This provides new research directions for exploring the molecular basis of MI and T2D comorbidity. The pathogeneses of these diseases is strongly affected by mitochondrial autophagy. At the molecular level, T2D involves complex interactions between genetic and environmental factors, and studies have explored the impact of mitochondrial dysfunction in T2D, suggesting that alterations in mitochondrial dynamics and mitophagy may be involved in disease development [18-20]. Mitophagy is a specialized type of autophagy that selectively removes damaged or dysfunctional mitochondria to maintain cellular homeostasis. Dysregulation of mitophagy has been linked to various pathological conditions, including neurodegenerative and metabolic disorders [21]. In MI, impaired mitophagy is related to increased oxidative stress and apoptosis in cardiomyocytes, potentially exacerbating cardiac injury [22]. Similarly, in T2D, defective mitophagy may lead to the accumulation of dysfunctional mitochondria, thereby contributing to insulin resistance and β-cell dysfunction [20]. The interplay between mitophagy and these diseases underscores the importance of understanding the molecular mechanisms governing this process.

We, therefore, aimed to clarify the differential expression of mitophagy-related genes in MI and T2D, and to understand how these differences might influence disease progression. First, we employed robust bioinformatics tools, including the R package GEOquery for data retrieval, limma for data normalization, and clusterProfiler for enrichment analysis. Second, we constructed protein-protein interaction (PPI) networks using STRING and Cytoscape. Third, we developed a risk-prediction model based on mitophagy-related differentially expressed genes (MRDEGs) using logistic and selection operator (LASSO) regression analyses. Finally, we integrated bioinformatics analyses to elucidate the effects of mitochondrial and immune cell infiltration on MI and T2D. When identifying genes closely associated with specific biological processes, such as mitophagy, from large-scale transcriptomic data, researchers face significant technical challenges. Variations in experimental platforms, tissue sources, and sample heterogeneity introduce substantial data biases, posing considerable demands on the accurate screening of MRDEGs. To ensure data comparability and result reliability, this study employed the R package sva for batch effect removal and utilized the limma package for data normalization, thereby minimizing inter-platform discrepancies. Additionally, we cross-referenced mitophagy-related gene sets from the GeneCards and MSigDB databases to enhance the specificity and comprehensiveness of our screening process. At the algorithmic level, we implemented a three-stage analytical pipeline integrating logistic regression, random forest, and LASSO regression: logistic regression was applied for initial screening, random forest for variable importance ranking, and LASSO regression for variable reduction and model optimization. This multi-algorithm approach enabled cross-validated identification of key MRDEGs. Through this integrated framework of multi-platform data processing and multi-algorithm screening, our study technically ensures the robustness of MRDEG identification and provides a solid data foundation for subsequent functional validation. The advantages of these methods include the ability to systematically analyze large datasets and identify potential biomarkers and targets for therapy. We aimed to determine the role of mitophagy in MI and T2D, which may pave the way for novel interventions to improve clinical outcomes.

## 2 MATERIALS AND METHODS

### 2.1 Data Download

Using the R package, GEOquery, the MI datasets GSE48060 and GSE29532 and the T2D datasets GSE76894 and GSE25724 was retrieved from the Gene Expression Omnibus (GEO) database. The R package sva was used to debatch the GSE48060 and GSE29532 datasets to obtain the integrated MI GEO datasets. The combined MI dataset included 80 MI and 27 control datasets. Similarly, the GSE76894 and GSE25724 datasets of T2D included 25 T2D and 91 control cases. The GeneCards database [23] (https://www.genecards.org/) and the Molecular Signatures Database (MSigDB) [24] (https://www.msigDB) were used to obtain MRGs [25]. After using the term “Mitophagy” as a search keyword and retaining only MRGs with “Protein Coding” and “Relevance Score > 1”, a total of 1,633 MRGs were obtained. Similarly, the reactome mitophagy gene set contained 29 MRGs by searching the MSigDB database using “Mitophagy” as the keyword. We obtained 1,633 MRGs after merging and removing duplicates.

Finally, the R package limma was used to normalize the combined MI and T2D datasets. The expression matrixes before and after removal were subjected to PCA to confirm the efficacy of batch effect correction.

### 2.2 Association of MI and T2D with MRDEGs

We divided the patients into disease and control groups based on the grouping of the combined MI and T2D datasets. Differential gene analysis between the disease and control groups was performed using the R package limma. We set |logFC| > 0 for the DEGs threshold. We constructed a volcano plot of the comparative analysis findings using the R package ggplot2. To obtain the MRDEGs associated with MI and T2D, combined MI and T2D datasets were obtained using variance analysis (|log FC| > 0). The DEGs associated with MRGs intersected and were mapped to Venn and MRDEGs.

### 2.3 GO Enrichment

GO analysis is a widely used statistical method for integrative functional enrichment studies [26]. We utilized the R package clusterProfiler to conduct GO enrichment analysis of MRDEGs, and used the Benjamini−Hochberg (BH) *p*-value correction method.

### 2.4 GSEA

GSEA is used to assess how genes are distributed in a predefined gene set within a ranked gene table based on their relationship with the phenotype, and thus determine their impact on the phenotype [27]. Genes in the combined MI and T2D datasets were ranked based on their logFC values. We used the R package clusterProfiler to conduct GSEA for the combined MI and T2D datasets.

### 2.5 PPI Network

We used the STRING database (https://cn.string-db.org/) to explore the interactions between established and predicted proteins, and constructed a PPI network related to MRDEGs. Genes within the PPI network that interacted with other genes were selected for subsequent analysis. The Cytoscape software was used to visualize the networks. The GeneMANIA database (https://genemania.org/) was used to create hypotheses regarding gene function, analyze gene lists, and prioritize genes for functional analyses. A PPI network was constructed by predicting genes functionally similar to MRDEGs using the GeneMANIA website.

### 2.6 Selection of Key Genes and Construction of the LASSO Risk Model

First, we performed logistic regression analysis according to the expression values of the MRDEGs in the combined MI datasets. Logistic regression analysis was used to analyze the relationship between independent and dependent variables. A forest plot was constructed to show the group expression of MRDEGs involved in this logistic regression model. Random Forest is an algorithm that combines multiple decision trees through ensemble learning. The random forest package was used to build a model based on the expression levels of MRDEGs. MRDEGs were screened using the random forest model to perform LASSO through the R package glmnet. The findings from the LASSO regression analysis are depicted using a diagnostic model map and a variable trajectory map. The results of the LASSO regression analysis were used to develop a diagnostic model for MI, and the MRDEGs included in the model were key genes. Finally, the LASSO risk score was calculated based on the LASSO regression analysis risk coefficients.

We substituted the expression levels of the key genes in the combined MI datasets into the LASSO risk score model to obtain a risk score. The MI group was divided into high- and low-risk groups based on the median risk score. Similarly, we substituted the key gene expression in the combined T2D datasets into the LASSO risk score model, and the T2D group was divided into high- and low-risk groups.

### 2.7 Diagnostic Performance of Key Genes and Validation of the Lasso Risk Model

The R package rms was used to create a nomogram using the outcomes from the LASSO regression analysis, illustrating how the expression of key genes relates to the diagnostic value in the MI and T2D combined datasets. We used the R package ggDCA to create DCA maps based on the key genes in the combined datasets. Finally, to assess the diagnostic value of the LASSO risk score on disease occurrence, we plotted the ROC curve and computed the area under the curve (AUC) of the LASSO RiskScore in the combined datasets using the R package pROC.

### 2.8 Verification of Differential Expression of Key Genes and ROC Curve Analysis

To further explore the differential expression levels of the key genes among the combined datasets, a group comparison map was drawn based on the expression levels of the key genes. Finally, the R package pROC was used to plot the ROC curve of the key genes and calculate the AUC to evaluate the diagnostic effect of the expression levels of the key genes on the occurrence of MI and T2D.

### 2.9 GSVA

We evaluated whether different pathways were enriched in the different samples. Gene sets were obtained from MSigDB [24], and two combined disease datasets were analyzed. All genes in the disease datasets were subjected to GSVA to calculate differences in functional enrichment between the high- and low-risk groups.

### 2.10 Immune Infiltration Analysis

In this study, we employed the CIBERSORT algorithm to analyze the composition of specific immune cell subsets in blood samples, aiming to explore the immune microenvironment characteristics in patients with MI and T2D. CIBERSORT is a deconvolution algorithm based on RNA-seq expression data, capable of quantifying the proportions of different immune cell types in mixed samples, thereby inferring the immune system alterations under disease conditions. Using the CIBERSORT algorithm combined with the immune cell signature gene matrix, and filtering out data with immune cell enrichment scores greater than zero, the specific results of the immune cell infiltration matrix in the disease were obtained, which are displayed in proportion bar charts. Subsequently, the relationship between immune cells and key genes was computed using Spearman’s algorithm.

### 2.11 Constructing the Regulatory Network

We used the ChIPBase database (http://rna.sysu.edu.cn/chipbase/) to retrieve TFs and analyze the regulation of key genes. Finally, an mRNA-TF regulatory network was created using Cytoscape. To analyze the interactions between key genes and miRNAs, we identified miRNAs that interacted with key genes from the ENCORI database [28]. Cytoscape was used to plot mRNA-miRNA interaction networks.

### 2.12 Statistical Analysis

All data processing and analyses were performed using R software (version 4.3.1). For comparisons of continuous variables between the two groups, the statistical significance of normally distributed variables was estimated using independent Student’s t-tests. The Mann–Whitney U Test (Wilcoxon rank-sum test) was used to analyze the differences between variables that were not normally distributed. The Kruskal–Wallis test was used to compare three or more groups. Spearman’s correlation analysis was used to calculate the correlation coefficients between different molecules. All statistical *p*-values were two-sided, and statistical significance was set at *p* < 0.05. To comprehensively identify genes potentially associated with these diseases, we applied an initial screening threshold of |logFC| > 0 and *p* < 0.05 in the differential expression analysis. This criterion was adopted to avoid excluding mitophagy-related genes with modest expression changes that may still hold biological significance, which could occur if a more stringent fold-change threshold were applied. Subsequently, a dual feature selection approach—integrating both Random Forest and LASSO regression—was employed for cross-validation. Random Forest was used to assess gene importance and capture potential nonlinear relationships, while LASSO regression was applied for feature compression and model simplification. This combined strategy leverages the complementary strengths of both algorithms, helping to reduce model bias and improve the robustness and interpretability of the final gene set. In this study, a relatively lenient initial screening threshold was employed in combination with a dual-algorithm cross-validation strategy to enhance the comprehensiveness of key gene identification and improve the stability of the model.

## 3 RESULTS

### 3.1 Technology Roadmap

A flow chart outlining the integrative analysis is presented in Fig. (**1**).

### 3.2 Identification of Genes Showing Differential Expression

First, we obtained the combined MI datasets using the R package sva to remove the batch effect on the MI datasets GSE48060 and GSE29532. Subsequently, distribution boxplots (Figs. **S1A** and **B**) were used to compare the values representing gene expression across the datasets. Second, we used a principal component analysis (PCA) plot (Figs. **S1C** and **D**) to compare the distribution of low-dimensional features before and after the batch effect was removed. The distribution boxplot and PCA plot results show that the batch effect in the combined MI datasets was eliminated by removing the batch. This was repeated for T2D datasets GSE76894 and GSE25724 (Figs. **S2A-D**). Altogether, we identified 1528 differentially expressed genes (DEGs; 734 upregulated and 794 downregulated genes) (Fig. **S3A**). The combined T2D datasets included 3,314 DEGs: 1,666 upregulated and 1,648 downregulated genes (Fig. **S3B**).

To obtain MI and T2D-related MRDEGs, intersection and map Venn diagrams were prepared (Fig. **S3C**). We identified 45 MI and T2D MRDEGs and analyzed the differential expression thereof among the different sample groups in the combined MI and T2D datasets (Figs. **S3D** and **E**). Finally, the locations of 45 MRDEGs on the human chromosome were analyzed (Fig. **S3F**). Chromosomal mapping revealed that more MRDEGs are located on chromosomes 6 and 17.

### 3.3 Functional Enrichment Analysis

The 45 MRDEGs were used for gene ontology (GO) analysis, and the findings are detailed in Table **S1**. MRDEGs were particularly enriched in the regulation of autophagy, mitochondrial autophagy, and mitochondrial disassembly in MI and T2D (Figs. **S4A-D**). We determine the effect of gene expression levels in the combined MI and T2D datasets by utilizing gene set enrichment analysis (GSEA). The molecular functions and all genes in the combined MI datasets that exhibited significant enrichment are shown in Figs. (**S5A-H** and **S6A-H**) and Tables **S2** and **S3**.

### 3.4 PPI Network Analysis

The STRING database was used to construct a PPI network of 45 MRDEGs (Fig. **2A**), showing that 29 MRDEGs were interconnected. Subsequently, an interaction network comprising 29 MRDEGs and functionally similar genes was identified (Fig. **2B**). Among these, 29 MRDEGs and 20 functionally similar proteins were identified.

### 3.5 Screening of Key Genes and Construction of the LASSO Risk Model

Logistic regression was performed using the expression levels of 29 MRDEGs from the combined MI datasets (Fig. **3A**). We identified 28 statistically significant MRDEGs. To assess the diagnostic value of the 28 MRDEGs, we employed the random forest algorithm to assess the expression levels of 28 MRDEGs (Figs. **3B**-**D**), and performed LASSO regression analysis. The results are shown in Figs. (**3E** and **F**). The nine key genes identified in the LASSO regression model were *ATG14, BIRC2, CSK, FBXO7, HNRNPH1, HNRNPL, SNX6, TFEB,* and *YES1*. Finally, based on the RiskScore formula, we substituted the expression levels of key genes in the combined MI and T2D datasets to obtain the RiskScore values thereof.

To integratively assess the diagnostic performance of key genes in the LASSO risk model, we created a logistic regression diagnostic model (Figs. **4A** and **B**). The points in the nomogram were assigned based on the coefficient weights of variables derived from the logistic regression model, which were linearly transformed to a 0-100 point scale. The results showed that the contribution of *ATG14* and *BIRC2* expression to MI diagnosis was markedly higher than that of other variables; however, the contribution of *TFEB* expression was significantly lower. The contribution of *CSK* expression to T2D diagnosis was significantly higher, and that of *FBXO7* expression to T2D diagnosis was significantly lower. The top axis of the nomogram corresponds to the expression level of each gene. To determine the contribution score (Points) for a specific gene, locate its actual expression value on the corresponding variable axis and project vertically downward to the Points scale. Higher expression levels of a gene generally result in higher assigned points. The points for all included genes are then summed to obtain the Total Points. This aggregate score is subsequently projected vertically downward to the bottom risk probability axis to determine the corresponding probability of disease occurrence. A higher Total Points value indicates a greater likelihood of the sample belonging to the disease group. We then performed a decision curve analysis (DCA) on the combined MI and T2D datasets to assess the clinical value of the key gene diagnostic models. The details are shown in Figs. (**4C** and **D**). Finally, the receiver operating characteristic (ROC) curve showed that the LASSO risk score in the combined MI datasets was accurate for MI diagnosis (Fig. **4E**; area under the curve [AUC] = 0.788). The T2DM results are presented in Fig. (**4F**) (AUC = 0.769).

### 3.6 Key Genes and Interactions

To investigate variations in the patterns of key gene expression across the combined MI and T2DM datasets, a group comparison was conducted. Figs. (**5A** and **B**) show the differential expression of nine key genes in both diseases. The differential outcomes (Fig. **5A**) indicated that three key genes, *HNRNPH1, SNX6,* and *TFEB*, were highly significant in the combined MI datasets. Similarly, the differential results (Fig. **5B**) showed that three key genes, *CSK, SNX6,* and *YES1*, were statistically significant in the combined T2D datasets. The ROC curve (Figs. **5C**-**E**) indicated that the expression levels of the key genes *ATG14, HNRNPH1, HNRNPL, SNX6, TFEB,* and *YES1* were accurate. Likewise, in the T2D datasets, the ROC curve (Figs. **5F**-**H**) suggested that the expression levels of the key genes *ATG14, CSK, SNX6,* and *YES1* exhibited better accuracy. To know the differences between the high and low-risk groups of these diseases, we utilized gene set variation analysis (GSVA) in the combined MI datasets (Table **S4** and Figs. **6A** and **B**), as well as in the T2D samples (Table **S5** and Figs. **7A** and **B**).

### 3.7 Immune Infiltration Analysis

The MI samples were used to assess the abundance of immune infiltration using the CIBERSORT algorithm. Fig. (**8A**) shows the proportion of immune cells in the MI samples. The results indicated the enrichment of 21 types of immune cells, and the correlation findings of the infiltration abundance are shown in Figs. (**8B** and **C**). CD4 naïve T cells and resting natural killer (NK) cells exhibited the most pronounced negative correlation with the low-risk MI group (Fig. **8B**), whereas resting and activated mast cells had the most significant negative relationship within the high-risk group (Fig. **8C**). The correlation between the key genes and immune cell infiltration abundance is presented in Figs. (**8D** and **E**). In the low-risk group, *TFEB* and CD4 memory resting T cells exhibited the strongest negative correlation (Fig. **8D**), whereas in the high-risk group, *ATG14* and neutrophils showed the strongest negative correlation (Fig. **8E**).

We also used the CIBERSORT algorithm to calculate immune cell infiltration in the combined T2D datasets (Figs. **9A**). The correlation results for these immune cell infiltration levels are shown in Figs. (**9B** and **C**). In the low-risk group, activated memory CD4^+^ and gamma delta T cells, as well as M2 macrophages and resting dendritic cells, exhibited the most significant positive correlation (Fig. **9B**). Among the high-risk groups, the activated NK cells and resting mast cells showed the most significant positive correlations (Fig. **9C**). *CSK* and activated NK cells exhibited the strongest negative correlation (Fig. **9D**), whereas *HNRNPL* and resting mast cells showed a positive relationship within the high- risk group (Fig. **9E**).

Using the ChIPBase database and Cytoscape software, based on the nine key genes (*ATG14, BIRC2, CSK, FBXO7, HNRNPH1, HNRNPL, SNX6, TFEB, YES1*), we constructed an mRNA-TF regulatory network (Fig. **10A**). Among these genes, seven key genes (*ATG14, BIRC2, CSK, FBXO7, SNX6, TFEB, YES1*) and 30 transcription factors constituted the mRNA-TF interaction relationships. Further details are presented in Table **S6**.

Using the ENCORI database, we obtained data related to nine key genes and the miRNAs predicted to interact with these genes, which were screened using Cytoscape (Fig. **10B**). Among them, seven key genes (*ATG14, BIRC2, CSK, HNRNPH1, HNRNPL, SNX6,* and *YES1*) and 46 miRNAs encompassing 48 mRNA-miRNA interaction pairs were identified.

The immune infiltration analysis revealed significant differences in the proportions of several immune cell types between the high-risk and low-risk groups, suggesting that MI and T2D may involve specific immune regulatory processes. This finding provides important insights for further investigating the role of the immune system in disease progression.

## 4 DISCUSSION

MI and T2D are formidable health challenges owing to their high global morbidity and mortality rates [2, 29]. The coexistence of MI and T2D is a common clinical condition. More than 40% of patients with MI have comorbid diabetes [30]. The treatment of these diseases relies primarily on medications tailored to their respective pathogenic mechanisms. Effective treatments for MI include percutaneous coronary intervention, thrombolytic treatment, and bypass surgery; however, these invasive approaches are unsuitable for all patients.

Another approach is pharmacotherapy, such as antiplatelet therapy, which has generally been proven to be inefficient owing to its non-targeted drug distribution and side effects [31]. The primary effective treatments for T2D include lifestyle interventions and pharmacotherapies. Glucose-lowering medications generally need to be taken long-term unless they are intolerable or cessation is strongly indicated owing to safety concerns arising from potential side effects [32]. The urgency to understand the common underlying molecular mechanisms of these two diseases is underscored by their increasing prevalence and substantial burden on healthcare systems [2, 29]. Therefore, there is increasing demand for safer and more efficient therapies and prevention strategies for MI and T2D.

Cardiac function is strongly dependent on mitochondrial oxidative metabolism and quality control [33]. Furthermore, mitophagy may contribute to the pathogenesis of MI and T2D [13, 18]; however, the effects of mitophagy on these diseases remain unknown. Our study addresses this critical need by elucidating the role of mitophagy-related genes (MRGs) in the pathogenesis of MI and T2D and provides insights that could pave the way for novel therapeutic strategies. Our integrative bioinformatics analysis revealed distinct patterns of MRG expression in MI and T2D, highlighting the value of these genes as biomarkers and therapeutic targets. GO enrichment and GSEA revealed previously unknown associations between MRGs and disease-specific pathways [27]. Furthermore, a predictive LASSO risk model based on MRG expression in patients with MI represents a significant advancement over existing diagnostic tools, offering improved risk stratification and personalized treatment approaches [34]. These findings, together with the identified novel mRNA-miRNA interaction networks, contribute substantially to the field by expanding our understanding of the molecular interplay between mitophagy and disease pathogenesis [35].

Through rigorous analysis, we identified nine genes (*ATG14, BIRC2, CSK, FBXO7, HNRNPH1, HNRNPL, SNX6, TFEB,* and *YES1*) that were significantly associated with MI and T2DM. *ATG14* is a crucial gene involved in the initiation of autophagy, particularly mitophagy, by forming a complex with Beclin-1 and VPS34, which are essential for autophagosome nucleation [36]. Hypoxia-induced decline in autophagy gene expression coincides with reduced *ATG14* protein levels [33]. *ATG14* expression is downregulated in patients with T2D [37]. *BIRC2*, also known as *cIAP1*, is an inhibitor of apoptotic proteins that plays a significant role in regulating cell death and survival pathways [38]. In the context of MI, *BIRC2* may help reduce cardiomyocyte apoptosis, thereby limiting the infarct size and improving cardiac function. Its activation induces apoptosis and cytoprotection. Upregulation of *BIRC2* expression can inhibit miR-205 expression, which may have an anti-apoptotic function [39]. Similarly, in T2D, *BIRC2* contributes to pathogenic mechanisms, such as lowered insulin production, development of insulin resistance, or diabetic nephropathy [40]. In MI, *CSK* influences cardiomyocyte survival and repair by regulating Src kinase activity. When *CSK*, a negative regulator of the Src family of tyrosine kinases, is overexpressed, the activation of transfected ERK2 with H_2_O_2_ is abolished. H_2_O_2_ induces apoptosis in cardiac myocytes [41]. A previous study indicated increased platelet aggregability and blood coagulability in diabetic participants compared to healthy volunteers, with *CSK2a* expression in the complex directly linked to these processes. The pathophysiology of diabetes involves high thrombotic risk and platelet reactivity [42]. *FBXO7* belongs to the F-box protein family and serves as a component that recognizes substrates within the SCF (SKP1-CUL1-F-box protein) ubiquitin ligase complex. This gene plays a crucial role in multiple cellular processes such as cell cycle regulation, apoptosis, and mitophagy [43]. *FBXO7*-mediated downregulation of cytoprotective SIRT7 may be closely associated with cell death [44]. Inhibition of *FBXO7* can disrupt the interaction between the mitochondrial kinase PINK1 and the ubiquitin machinery, which can effectively suppress inflammation, thereby enhancing mitochondrial quality [45]. However, further research is required to clarify the precise prognostic significance of *FEBXO7* in patients with diabetes. In MI, *TFEB* protects cardiomyocytes from ischemia/reperfusion (I/R) injury by enhancing mitochondrial quality control [46, 47]. *TFEB* promotes the transcription of genes that regulate various phases of autophagy. Glucolipotoxicity suppresses *TFEB* expression in cardiomyocytes and inhibits lysosomal autophagy in obesity and diabetes [47]. *TFEB* expression is reportedly reduced in the nucleus during reperfusion compared to that in the sham and ischemia groups [47]. *TFEB* enhances autophagy in the heart during I/R [48], and independent studies have shown that *TFEB* activation protects against the effects of metabolic syndromes by increasing the capacity of cells to upregulate autophagy [49]. *YES1* belongs to the Src family of tyrosine kinases and plays a crucial role in many cellular processes such as proliferation, differentiation, and survival [50]. *YES1* expression is significantly increased in myocardial I/R injury models compared to that in control groups. Overexpression of miR-140 inhibits mitochondrial apoptosis induced by myocardial I/R by targeting *YES1,* which reduces myocardial I/R-related increases in infarct size and myocardial enzymes [51]. The expression level of *IFIH1* was significantly correlated with that of *YES1*, a kinase that participates in GLUT4-mediated glucose transport. Variations in *YES1* polymorphisms among individuals correlate with different levels of *IFIH1*, indicating that *IFIH1* influences susceptibility to diabetes by modulating glucose transport mechanisms [52].

We constructed a multivariable nomogram involving all nine hub MRDEGs to enhance the practical use of the theoretical diagnostic genome in routine clinical conditions. We quantified the expression of each gene and transformed it into a score that subsequently correlated with a linear predictor. Furthermore, GSEA identified remarkable enrichment in pathways such as TGF-beta receptor signaling and the IL-12 STAT4 pathway. The TGF-beta signaling pathway is involved in cardiac remodeling and fibrosis, which are crucial processes in MI progression [53]. Similarly, the IL-12 STAT4 pathway plays a significant role in immune response modulation, which is pertinent given the inflammatory nature of both MI and T2D [54]. The involvement of these pathways suggests that dysregulation of MRGs may contribute to the pathological remodeling and immune responses observed in these diseases.

Immune infiltration typically refers to the presence of immune cells in specific tissues or body fluids (such as blood), used to assess the activation status of the immune system and its potential role in disease. Mitophagy is a vital form of autophagy for the selective removal of dysfunctional or redundant mitochondria. Accumulating evidence implicates the elimination of dysfunctional mitochondria as a powerful means employed by autophagy to keep the immune system in check. The process of mitophagy may restrict inflammatory cytokine secretion and directly regulate mitochondrial antigen presentation and immune cell homeostasis [55]. Canonical autophagy is the basis of mitophagy, which can be mechanistically divided into multiple steps involving the coordination of various autophagy-related (ATG) proteins [56]. In addition, ATG14, which is well-known for its role in autophagosome initiation, also directly binds to the STX17-SNAP29 binary t-SNARE complex to promote its fusion with lysosomal VAMP8 [57]. STX17 recruits ATG14 and, subsequently, the core autophagy proteins to the mitochondria and MAM to form mitophagosomes [58]. Previous studies have revealed that *BIRC2* plays a critical molecular link between mitophagy and apoptosis [59]. FBXO7 plays a crucial role in the regulation of important mitochondrial events such as mitophagy and the protea some process of protein quality control [60-62]. Immune infiltration analysis revealed significant differences in immune cell infiltration patterns between the low- and high-risk groups, particularly highlighting a strong inverse relationship between *TFEB* and resting memory CD4^+^ T cells. *TFEB* is linked to a range of cellular processes, including immune responses [63]. The negative correlation with resting memory CD4^+^ T cells suggests that *TFEB* may influence the adaptive immune response, potentially affecting chronic inflammation observed in patients with MI and T2D. This observation is crucial, as it underscores the potential effect of immune modulation on the pathogenesis and progression of MI and T2D. This is supported by the lock-mediated enhancement of *TFEB* promoter activity in hypoxic cardiac myocytes expressing Clock [33]. *TFEB* inactivation in T cells leads to hyperimmunoglobulin M syndrome, primarily because CD4^+^ T cells show reduced expression of the CD40 ligand. TFEB directly stimulates Cd40lg expression in activated CD4^+^ T cells and plays a significant role in T cell-dependent antibody responses [64].

This study has several strengths. First, we identified MRDEGs, and integrative bioinformatic analysis enabled us to use genetic data to explore the association between MI and T2D. Second, our study highlights the significance of immune cell infiltration in the context of MI and T2D, offering new insights into disease mechanisms and potential avenues for therapeutic interventions.

## 5 STUDY LIMITATIONS

This study also has some limitations. Although our research does not fundamentally alter existing prevention or treatment approaches, it has the potential to enhance them. Our study lacked integration with laboratory-based experiments. Although bioinformatics analyses provide valuable insights, experimental validation is crucial to confirm these findings. A further limitation is that the sample sizes of the datasets used may have been relatively limited, which may have affected the robustness and generalizability of the results. To mitigate this, we integrated multiple independent GEO datasets and applied the sva package for batch effect correction and the limma package for normalization, thereby reducing systematic biases introduced by sample heterogeneity. Furthermore, we employed a comprehensive set of robust bioinformatic methods—including differential expression analysis, functional enrichment, random forest, and LASSO regression modeling—and validated model stability and predictive performance through cross-validation and ROC curve analysis. These approaches collectively help alleviate the impact of the limited sample size. In future work, we plan to expand the cohort size and incorporate experimental studies to perform multi-center validation of key genes and models, thereby enhancing the reliability and generalizability of our conclusions. The lack of clinical validation further limits the applicability of these findings to real-world scenarios. Although this study was conducted using data from the GEO database and a robust predictive model was constructed, we acknowledge that the absence of a validation cohort may impact the generalizability of the results. Currently, our analysis is primarily based on publicly available datasets, and future research will incorporate independent patient cohorts to more accurately assess the role of mitochondrial autophagy in the comorbidity of MI and T2DM. Finally, the use of multiple datasets may have introduced batch effects, which could potentially confound the results despite normalization efforts.

## CONCLUSION

Our research provides a bioinformatics analysis of MRGs. The screened genes, *ATG14, BIRC2, CSK, FBXO7, HNRNPH1, HNRNPL, SNX6, TFEB,* and *YES1*, may be therapeutic targets to prevent progression and improve clinical outcomes in patients with MI and T2D. Future studies should pay attention to validating these findings in a larger population and exploring the therapeutic prospects of modulating mitophagy and immune responses in these diseases.

## Figures and Tables

**Fig. (1):**
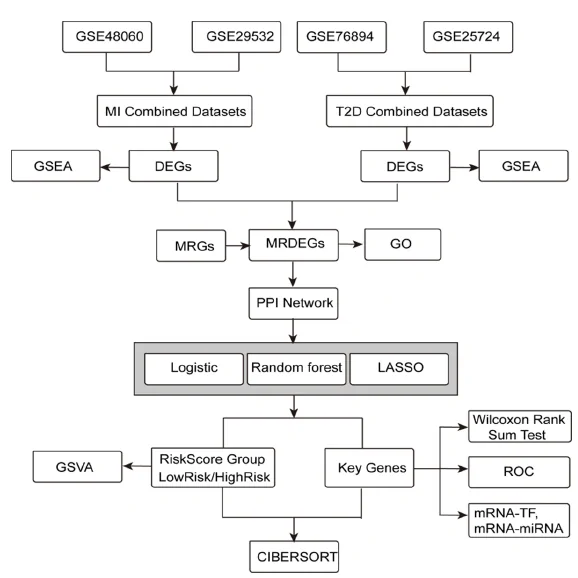
Flow chart of the integrative analysis. **Abbreviations:** MI, myocardial infarction; T2D, type 2 diabetes; DEGs, differentially expressed genes; MRGs, mitophagy-related genes; MRDEGs, mitophagy-related differentially expressed genes; ROC, receiver operating characteristic; GSEA, gene set enrichment analysis; GSVA, gene set variation analysis; PPI, protein-protein interaction; GO, gene ontology; LASSO, least absolute shrinkage and selection operator; TF, transcription factor.

**Fig. (2):**
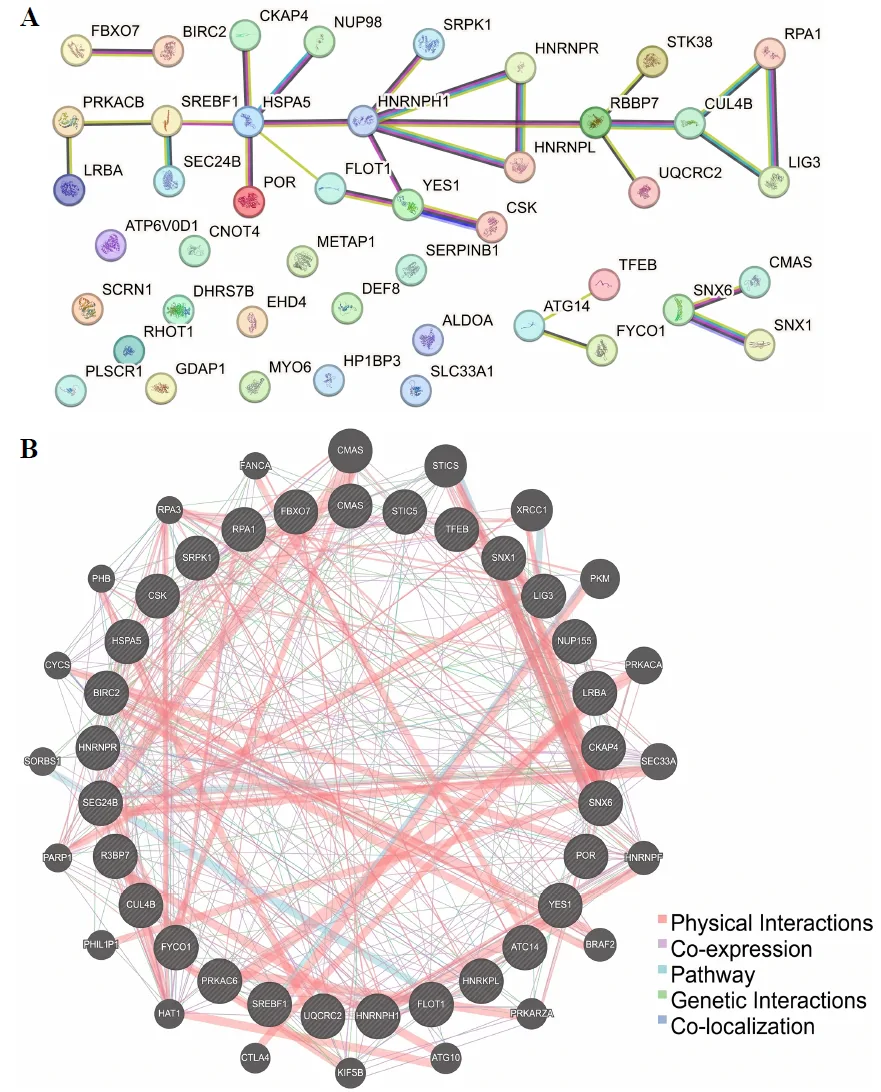
Protein-protein interaction (PPI) network analysis. (**A**) PPI network of mitophagy-related differentially expressed genes (MRDEGs) obtained from the STRING database. (**B**) The interaction network of functionally similar genes of MRDEGs is predicted by the GeneMANIA website. The circles in the figure show the MRDEGs and their functionally similar genes in our study, and the corresponding colors of the lines represent the interconnected functions.

**Fig. (3):**
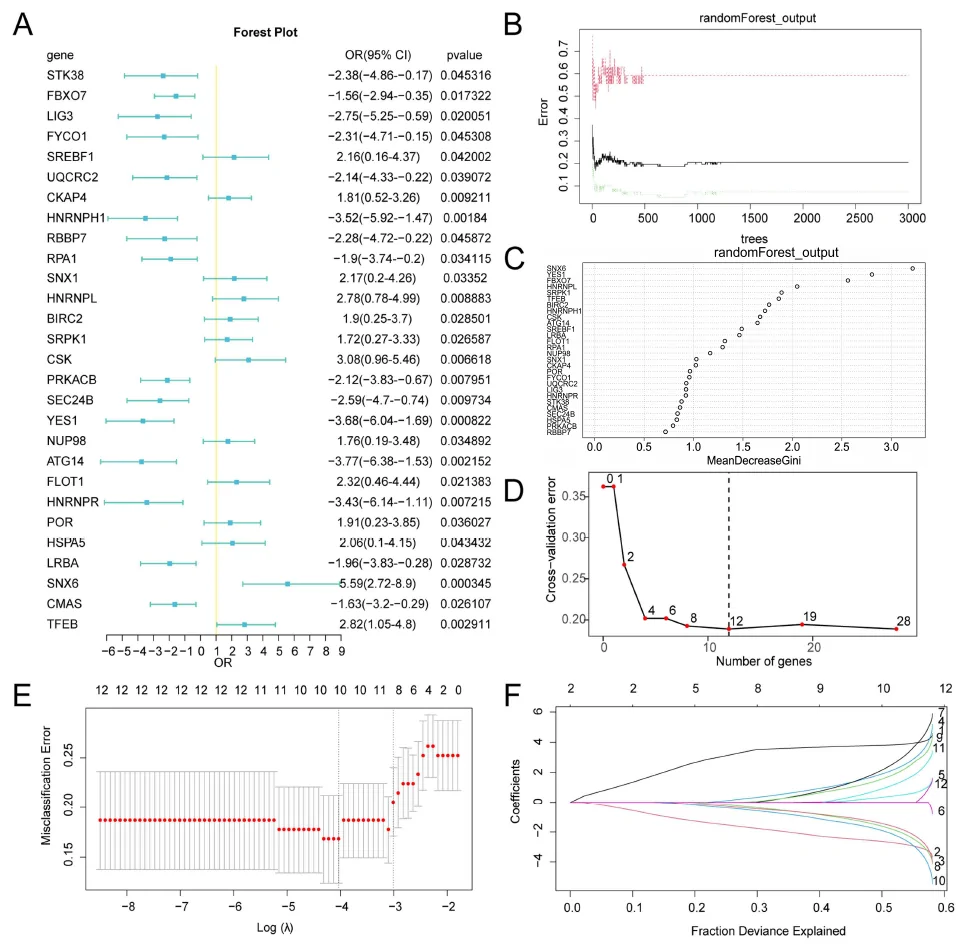
Diagnostic model of the combined myocardial infarction (MI) datasets. (**A**) Forest plot of 28 statistically significant (*p* < 0.05) mitophagy-related differentially expressed genes (MRDEGs) included in the logistic regression model for MI diagnosis. (**B**) Plot of model training error of the random forest algorithm. (**C**) MeanDecreaseGini scatter plot (descending MeanDecreaseGini order). (**D**) Cross-validation error plot. (**E**) Diagnostic plot of the least absolute shrinkage and selection operator (LASSO) regression model. (**F**) Variable trajectory plot of the LASSO regression model.

**Fig. (4):**
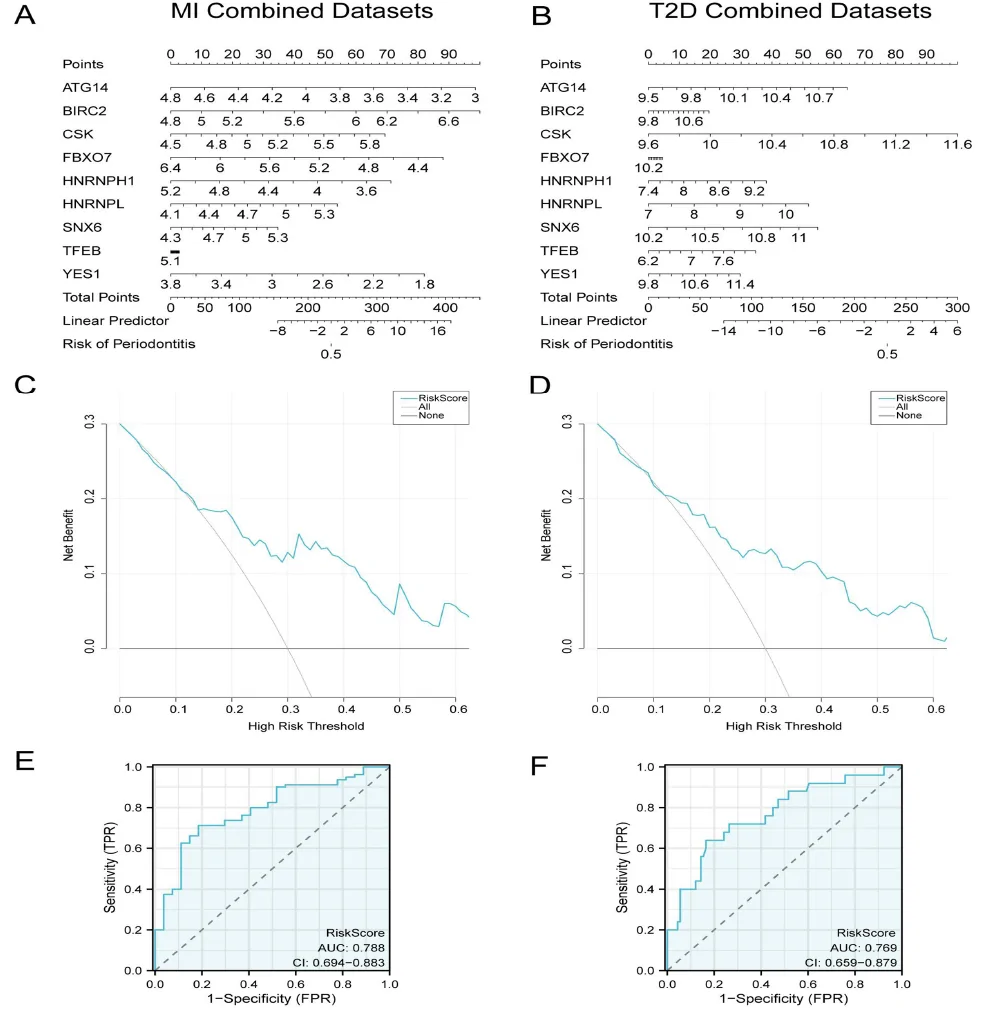
Diagnostic performance of key genes and validation of the least absolute shrinkage and selection operator (LASSO) risk model. (**A**) Nomogram of key genes in the myocardial infarction (MI) LASSO risk diagnostic model based on the combined MI datasets. (**B**) Nomogram of key genes based on the combined type 2 diabetes mellitus (T2DM) datasets in the T2D LASSO risk diagnosis model. (**C**) Decision curve analysis (DCA) plot of the risk score (RiskScore) of the LASSO risk diagnosis model for MI based on the combined MI datasets. (**D**) DCA plot of the risk score (RiskScore) of the LASSO risk diagnosis model for T2D based on the combined T2D datasets, where the ordinate of the DCA plot indicates the net benefit, and the abscissa indicates the probability threshold or threshold probability. (**E**) Receiver operating characteristic (ROC) curves of RiskScore from LASSO in the combined MI datasets. (**F**) ROC curves of the LASSO risk score (RiskScore) in the combined T2D datasets. The closer the area under the curve (AUC) is to 1, the better the diagnostic effect. The AUC has a certain accuracy in the range of 0.7−0.9. **Abbreviations:** TPR, true positive rate; FPR, false positive rate.

**Fig. (5):**
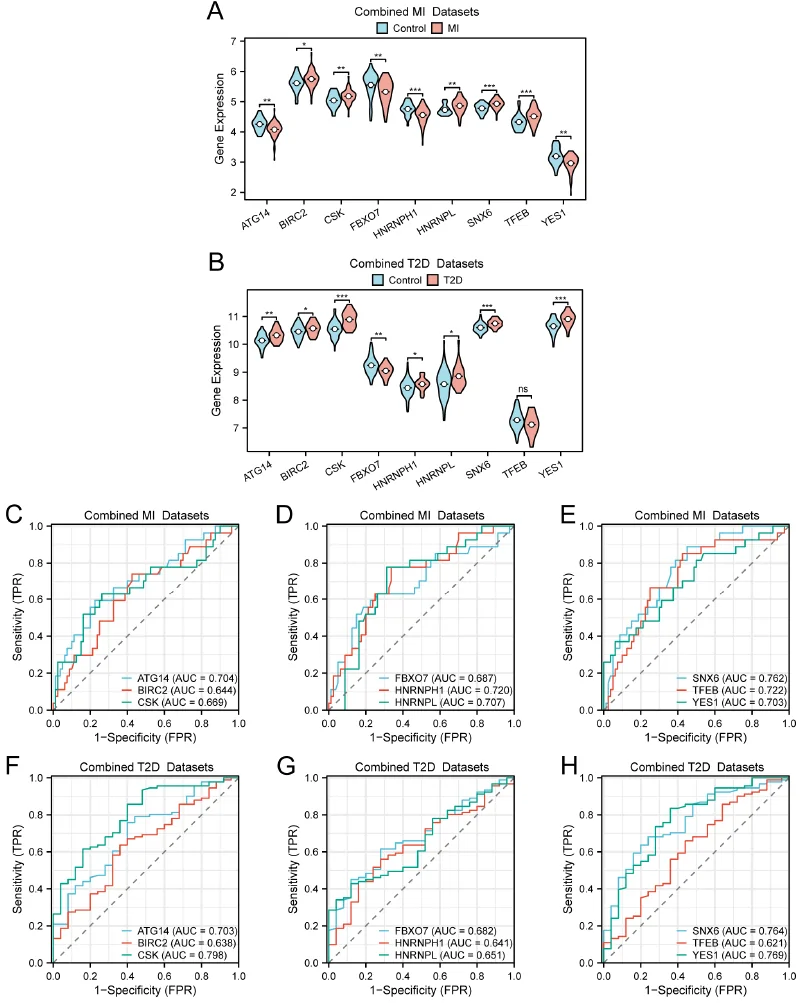
Differential expression validation and receiver operating characteristic (ROC) curve analysis. (**A**) Group comparison plots of key genes in the myocardial infarction (MI) and control (Control) groups of the combined MI datasets. (**B**) Group comparison plots of key genes in the type 2 diabetes mellitus (T2DM) and control groups of the combined T2D datasets. ROC curves of the *ATG14*, *BIRC2*, *CSK* (**C**), *FBXO7*, *HNRNPH1*, *HNRNPL* (**D**), *SNX6*, *TFEB*, and *YES1* (**E**), key genes in the combined MI datasets. ROC curves of the *ATG14*, *BIRC2*, *CSK* (**F**), *FBXO7*, *HNRNPH1*, *HNRNPL* (**G**), *SNX6*, *TFEB*, and *YES1* (**H**), key genes in the combined T2D datasets. ns, not statistically significant (*p* ≥ 0.05); *p < 0.05, statistically significant; **p < 0.01, highly statistically significant; ***p < 0.001, highly statistically significant. An area under the curve (AUC) > 0.5 indicates that the expression of the molecule tends to promote the occurrence of the event, and the closer the AUC is to 1, the better the diagnostic effect. An AUC between 0.5 and 0.7 indicated low accuracy, and between 0.7 and 0.9 indicated moderate accuracy. Red represents the disease groups (MI and T2D groups), and blue represents the control (Control) group. **Abbreviations:** TPR, true positive rate; FPR, false positive rate.

**Fig. (6):**
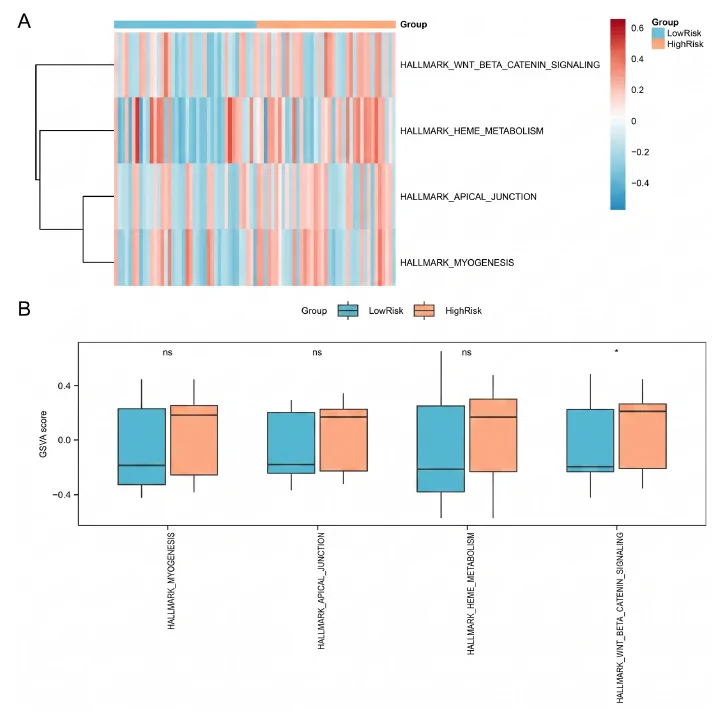
Gene set variation analysis (GSVA). Heat map (**A**) and group comparison map (**B**) of GSVA results between the high (HighRisk) and low-risk (LowRisk) groups of myocardial infarction (MI) samples from the combined MI datasets. ns, not statistically significant (*p* ≥ 0.05); *p < 0.05, statistically significant. Red represents the HighRisk group, and blue represents the LowRisk group. The screening criterion for GSVA was *p* < 0.05. In the heat map, blue represents low enrichment, and red represents high enrichment.

**Fig. (7):**
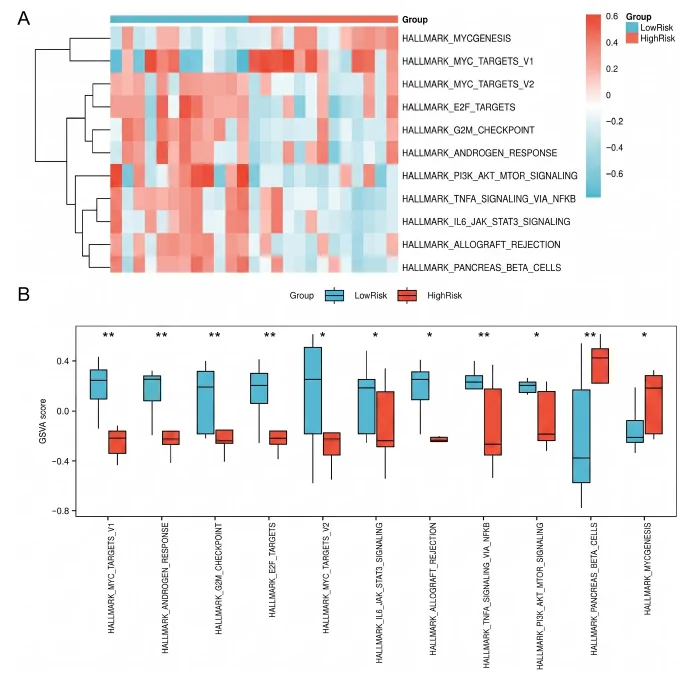
Gene set variation analysis (GSVA). Heat map (**A**) and group comparison map (**B**) of GSVA results between the high-risk (HighRisk) and low-risk (LowRisk) groups of type 2 diabetes mellitus (T2DM) disease samples in the combined T2D datasets. *p < 0.05, statistically significant; **p < 0.01, highly statistically significant. Red represents the HighRisk group, and blue represents the LowRisk group. The screening criterion for GSVA was *p* < 0.05. Blue represents low enrichment, and red represents high enrichment in the heat map.

**Fig. (8):**
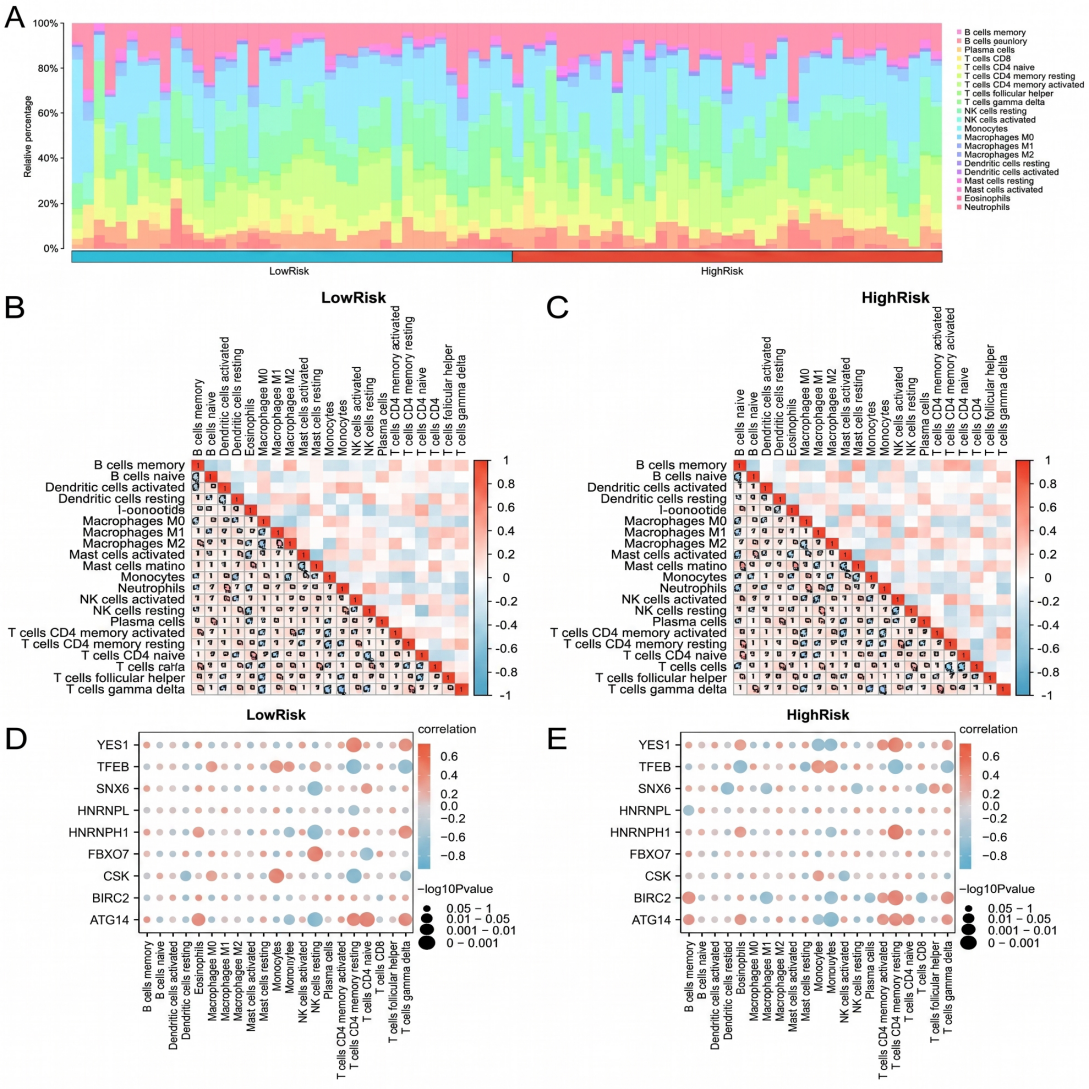
Risk group immune infiltration analysis using the CIBERSORT algorithm. (**A**) Bar chart showing the proportion of immune cells in myocardial infarction (MI) samples. Correlation heat map of immune cells in the low-risk (**B**) and high-risk (**C**) groups of MI samples. Bubble plot of correlation between immune cell infiltration abundance and key genes in low-risk (**D**) and high-risk (**E**) MI samples. An absolute correlation coefficient (r value) < 0.3 indicates weak or no correlation; 0.3−0.5, weak correlation; 0.5−0.8, moderate correlation; and > 0.8, strong correlation. Blue represents the low-risk group, and red represents the high-risk group. Red dots indicate a positive correlation, and blue dots indicate a negative correlation. The color intensity indicates the strength of the correlation.

**Fig. (9):**
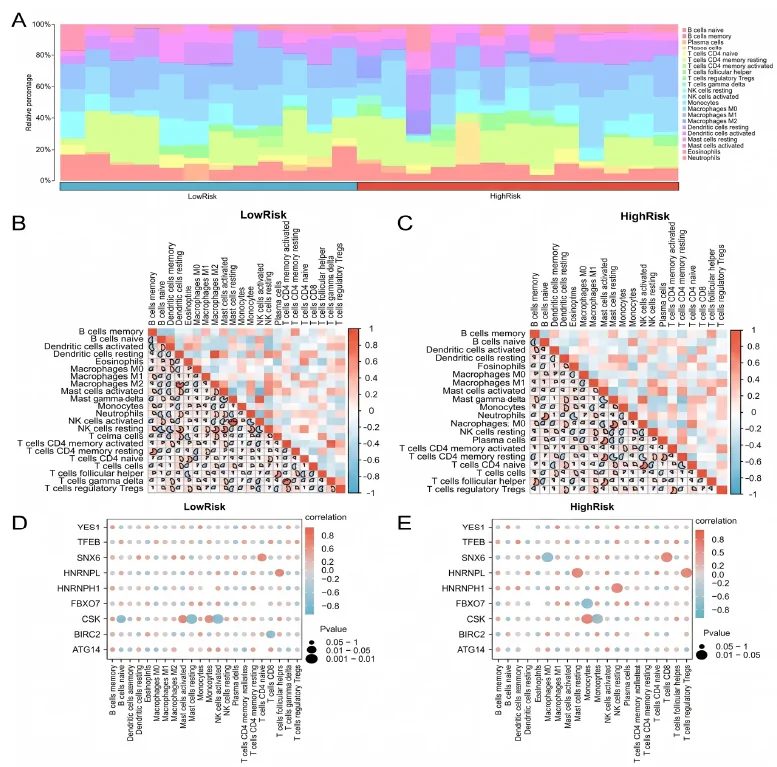
Risk group immune infiltration analysis using the CIBERSORT algorithm. (**A**) Bar chart of the proportion of immune cells in type 2 diabetes mellitus (T2DM) samples. Heat map of the correlation of immune cells in the low-risk (**B**) and high-risk (**C**) groups of T2D samples. Bubble plot of correlation between immune cell infiltration abundance and key genes in the low-risk (**D**) and high-risk (**E**) groups of T2D samples. An absolute correlation coefficient (r value) < 0.3 indicates weak or no correlation; 0.3−0.5, weak correlation; 0.5−0.8, moderate correlation; and > 0.8, strong correlation. Blue denotes the low-risk group, while red denotes the high-risk group. Red indicates a positive correlation; blue indicates a negative correlation. The color intensity represents the strength of the correlation.

**Fig. (10):**
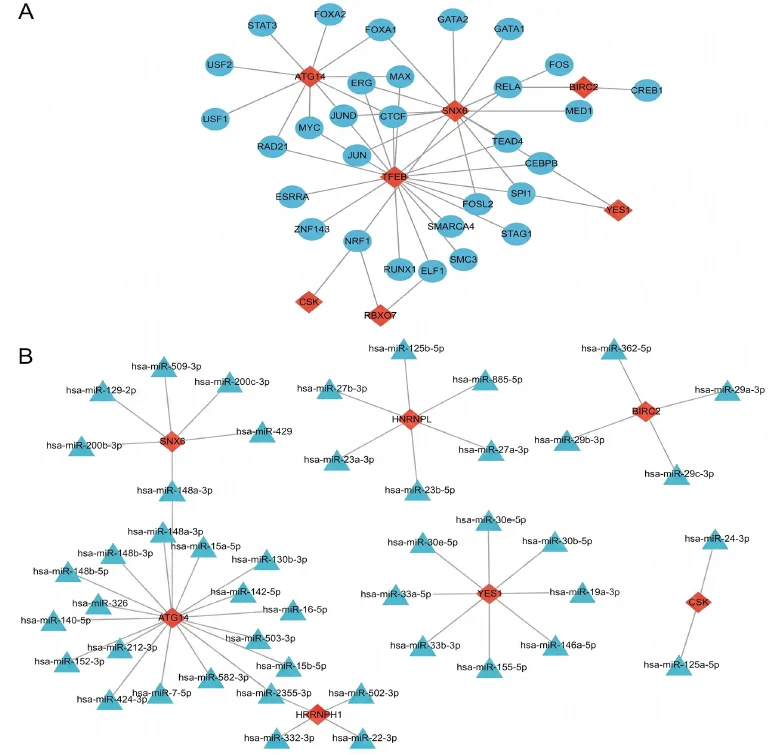
Regulatory network analysis of key genes. (**A**) mRNA-transcription factor (TF) regulatory network of key genes. (**B**) mRNA-miRNA regulatory network of key genes. Red diamonds indicate mRNAs, blue circles indicate TFs, and blue diamonds indicate miRNAs.

## Data Availability

The datasets used or analyzed in the current study are available from the corresponding author upon reasonable request.

## LIST OF ABBREVIATIONS

MI: Myocardial Infarction
T2DM: Type 2 Diabetes Mellitus
MRDEGs: Mitophagy-related Differentially Expressed Genes
CVD: Cardiovascular Disease
PPI: Protein-protein Interaction
