# Supplementary material for: An Integrative Analysis of the Role of Mitophagy in Comorbid Myocardial Infarction and Type 2 Diabetes Mellitus
Source: Endocr Metab Immune Disord Drug Targets. 2025 Apr 8;26:E18715303431252. doi: 10.2174/0118715303431252260315125017 (PMC13670198; doi:10.2174/0118715303431252260315125017)
Supplement: Supplementary file 1 [file EMIDDT-26-E18715303431252_SD1.pdf]

Supplementary Material

An Integrative Analysis of the Role of Mitophagy in Comorbid Myocardial Infarction and Type 2 Diabetes Mellitus

Ying Yang[1], Feiyan Wan[2], Xuelian Xu[3], Wei Xu[1], Lingli Jiang[4] and Pei Pan[4,*]

[1]Department of Cardiology, Chinese Academy of Medical Sciences and Peking Union Medical College, Fuwai Hospital; Beijing, 100037, China; [2]Department of Neurology, Guangzhou Eighth People's Hospital, Guangzhou Medical University. No.8,Huaying Road, Jiahe Street, Baiyun District, Guangzhou City, Guangdong Province, 510440, China; [3]Department of Special Intensive Care Unit, The First Branch of the First Affiliated Hospital of Chongqing Medical University, Chongqing, 400042, China; [4]Department of Cardiology, University-Town Hospital of Chongqing Medical University, Chongqing, 401331, China

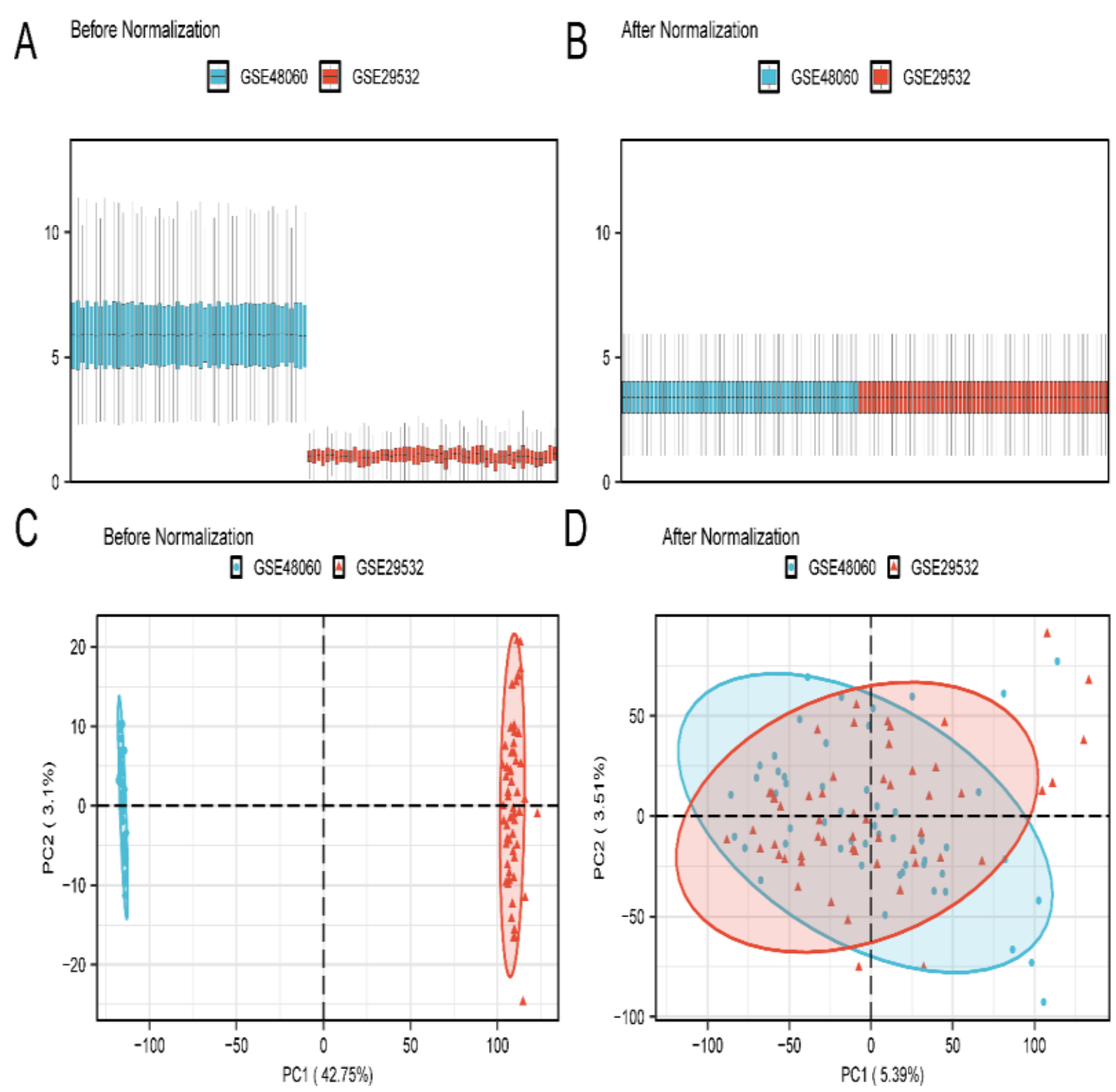

Figure S1. Removal of batch effects on GSE48060 and GSE29532. (A) Boxplot of the combined MI dataset distribution before removal of the batch effect. (B) Boxplot of the combined MI dataset distribution after removal of the batch effect. (C) PCA plot of the datasets before debatching. (D) PCA plot of the combined MI datasets after batching. The MI dataset GSE48060 is shown in blue, and the MI dataset GSE29532 is shown in red. PCA, principal component analysis; MI, myocardial infarction.

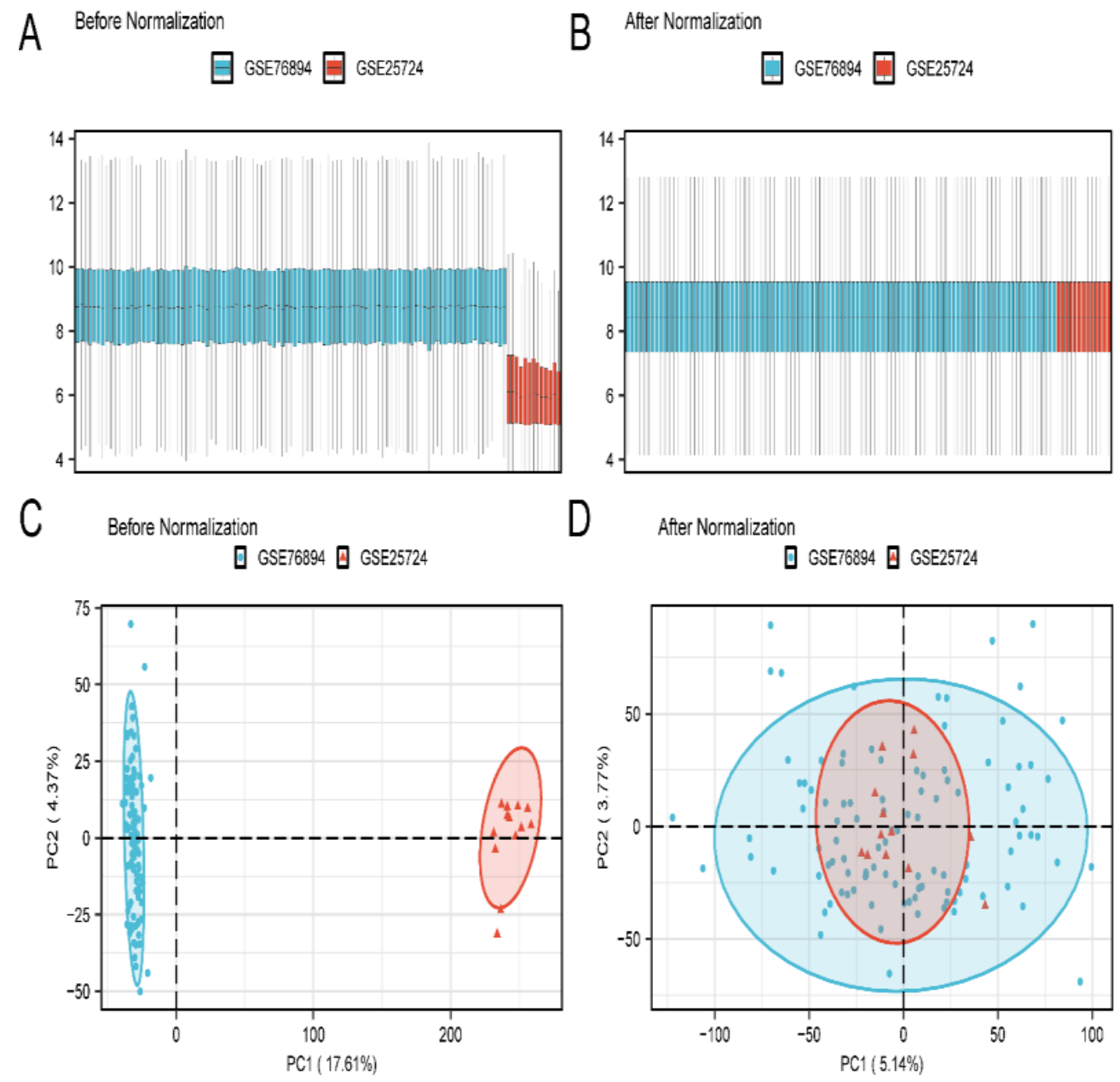

Figure S2. Removal of batch effects on GSE76894 and GSE25724. (A). Boxplot of the combined T2D dataset distribution before removal of the batch effect. (B) Boxplot of the combined T2D dataset distribution after removal of the batch effect. (C) PCA plot of datasets before debatching. (D) PCA plot of the combined T2D datasets after batching. The T2D dataset GSE76894 is shown in blue, and the T2D dataset GSE25724 is shown in red. PCA, principal component analysis; T2D, type 2 diabetes.

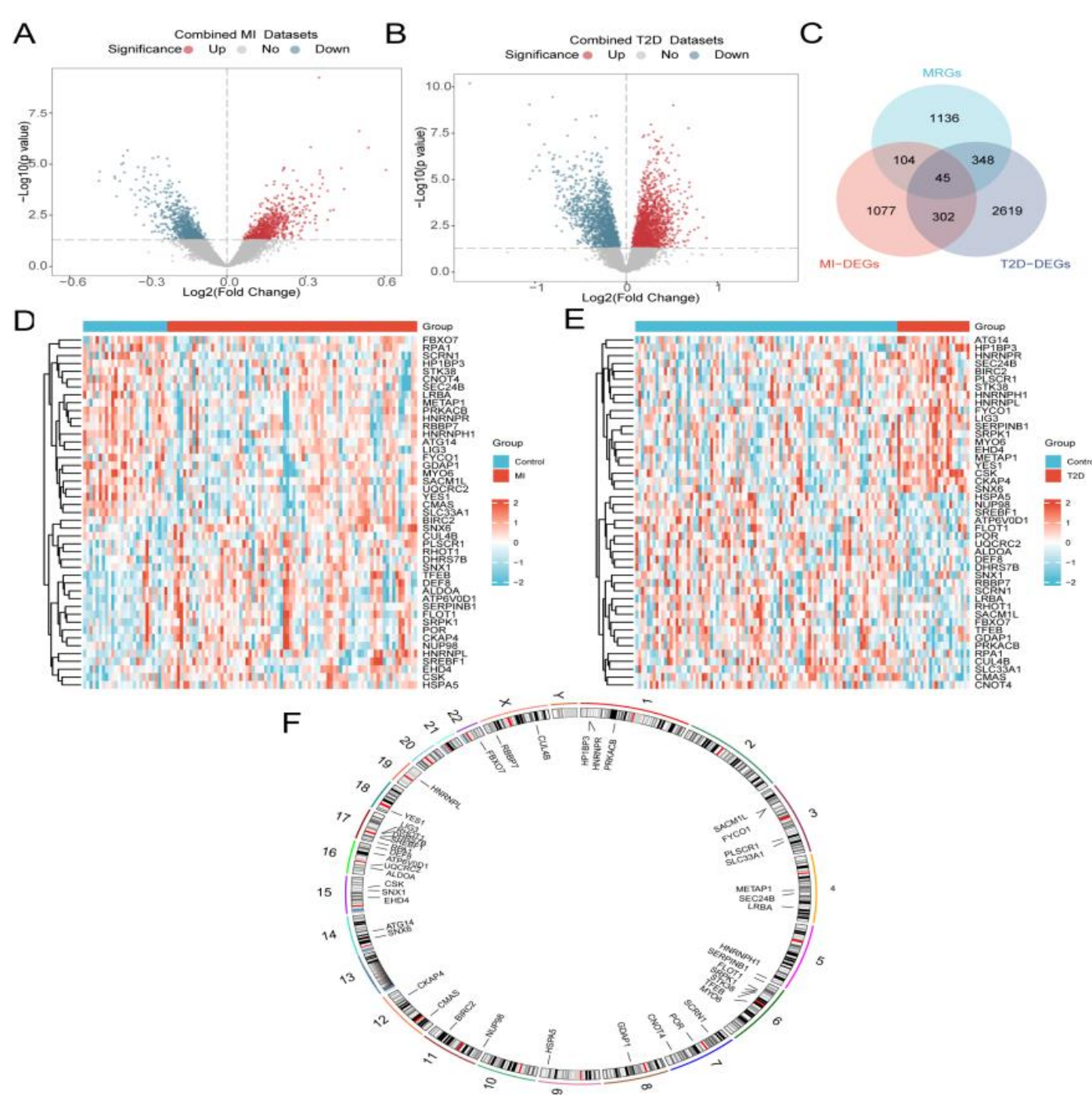

Figure S3. Differential gene expression analysis. (A) Volcano plot of the analysis of differentially expressed genes (DEGs) between myocardial infarction (MI) and control (Control) groups in the combined MI datasets. (B) Volcano plot of the analysis of DEGs between the type 2 diabetes mellitus (T2D) and control (Control) groups in the combined T2D datasets. (C) Venn diagram of DEGs and mitophagy-related genes (MRGs) in the combined MI and T2D datasets. (D) Heat map of mitophagy-related differentially expressed genes (MRDEGs) in the combined MI datasets. (E) Heat map of MRDEGs in the combined T2D datasets. (F) Chromosomal mapping of MRDEGs. Red indicates the disease group (MI, T2D groups), and blue indicates the control group. In the heat map, red represents high expression, and blue represents low expression.

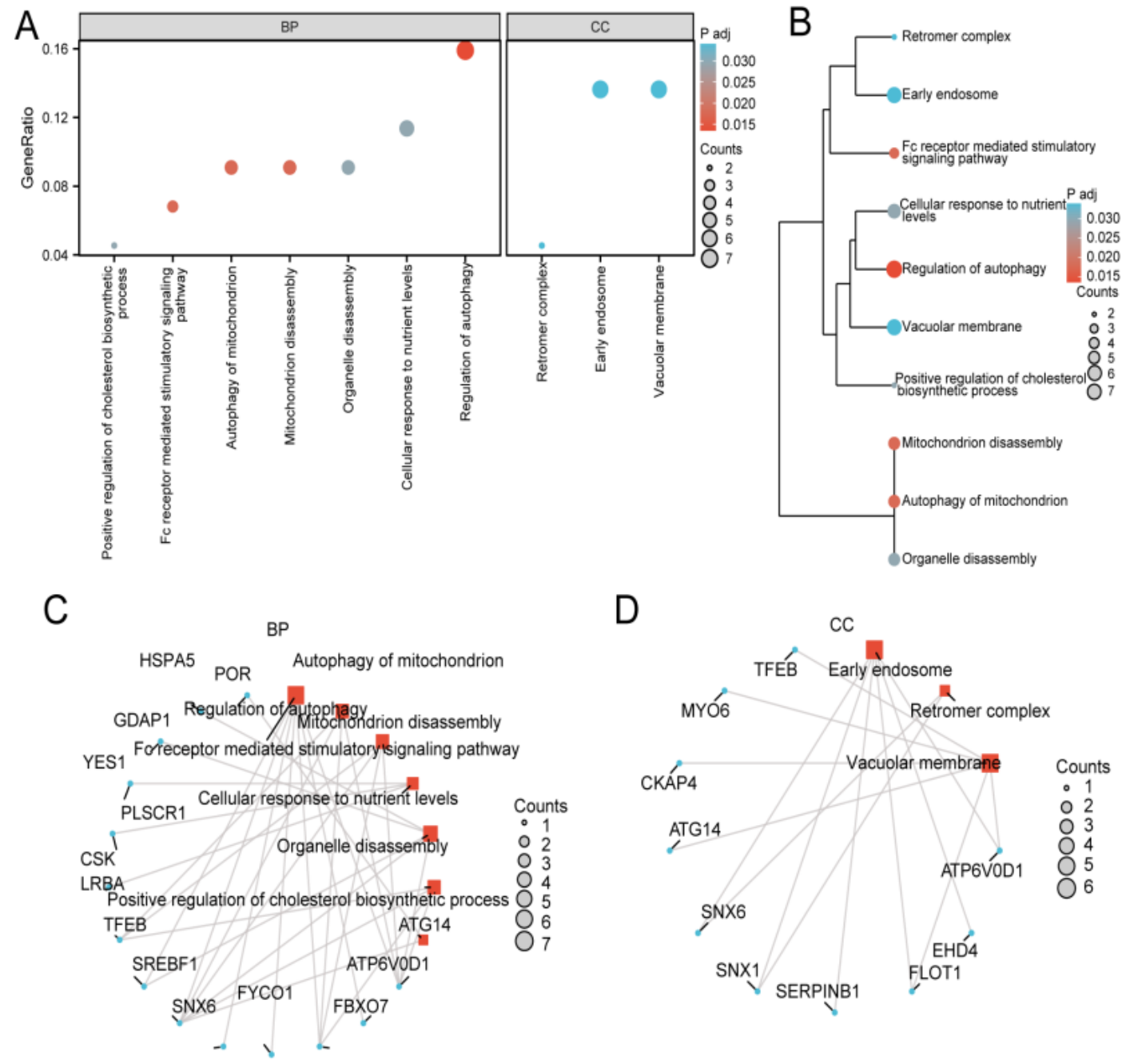

Figure S4. Gene ontology (GO) enrichment analysis for mitophagy-related differentially expressed genes (MRDEGs). (A) Bubble plot of GO enrichment analysis results of MRDEGs: biological process (BP), cellular component (CC). GO and Kyoto Encyclopedia of Genes and Genomes terms are shown on the abscissa. (B) GO enrichment analysis of MRDEGs. Cluster map of enrichment analysis results. (C-D) Network diagram of GO enrichment analysis results of MRDEGs: BP (C), CC (D). The bubble size in the bubble plot represents the number of genes, and the bubble color represents the size of the adjusted p value; the redder the color, the smaller the adjusted p value, and the bluer the color, the larger the adjusted p value. The screening criteria for GO enrichment analysis was adjusted p < 0.05 and false discovery rate (FDR) (q value) < 0.05 (statistically significant). p-value correction was done using the Benjamini−Hochberg (BH) method.

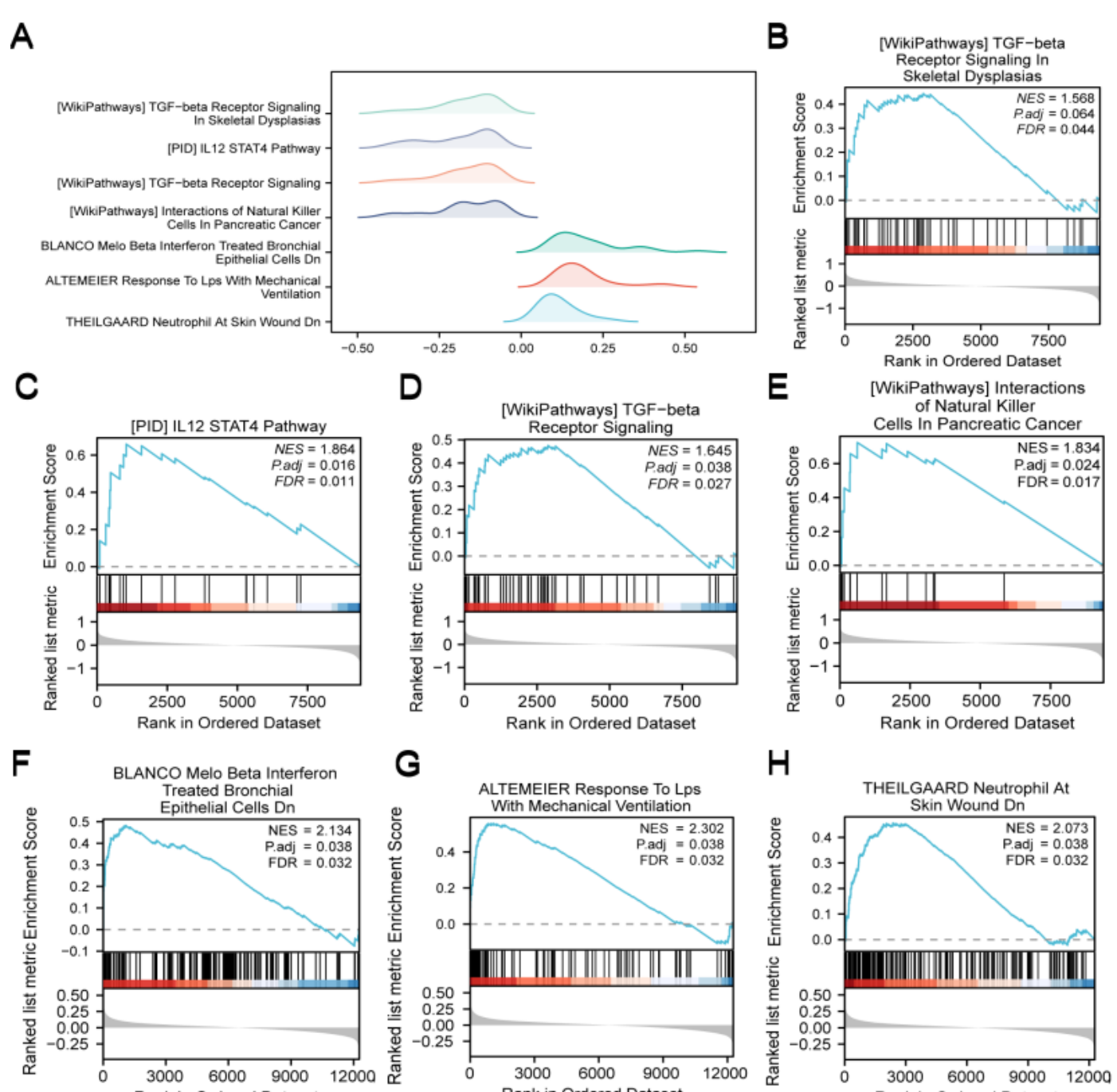

Figure S5. Gene set enrichment analysis (GSEA) of the combined myocardial infarction (MI) datasets. (A) Seven biological function mountain map presentations of the combined MI datasets. (B-H) GSEA showed that all genes were significantly enriched in TGF-beta receptor signaling in skeletal dysplasias (B), IL-12 STAT4 pathway (C), TGF-beta receptor signaling (D), interactions of natural killer cells in pancreatic cancer (E), BLANCO MELO beta interferon treated bronchial epithelial cells ductal normal (DN) (F), Altemeier response to lipopolysaccharide (LPS) with mechanical ventilation (G), and Theilgaard neutrophil at skin wound DN (H). The screening criteria for GSEA were adjusted p < 0.05 and FDR (q value) < 0.05. We used the Benjamini−Hochberg (BH) p-value correction method.

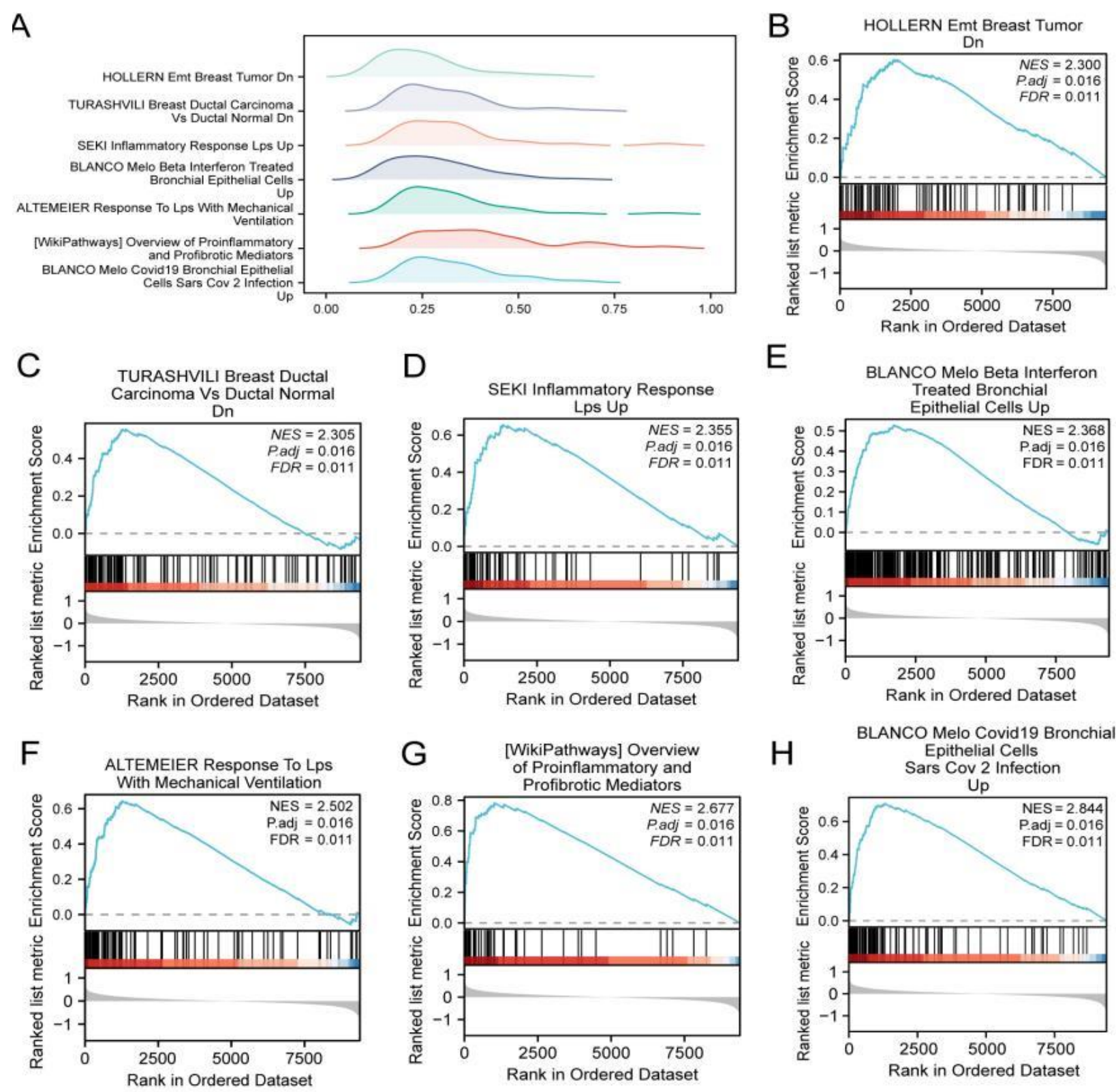

Figure S6. Gene set enrichment analysis (GSEA) for the combined type 2 diabetes mellitus (T2D) datasets. (A) Presentation of seven biological function mountain maps for GSEA of the combined T2D datasets. (B-H) GSEA showed that all genes were significantly enriched in Hollern EMT breast tumor ductal normal (DN) (B) Turashvili breast ductal carcinoma vs. DN (C), SEKI inflammatory response lipopolysaccharide (LPS) UP (D), BLANCO MELO beta interferon treated bronchial epithelial cells UP (E), Altemeier response to LPS with mechanical ventilation (F), WikiPathways; overview of proinflammatory and profibrotic mediators (G), BLANCO MELO coronavirus disease 2019 (COVID-19) bronchial epithelial cells severe acute respiratory syndrome coronavirus (SARS COV) infection UP 2 (H). The screening criteria for GSEA were adjusted $p < 0.05$ and FDR value (q value) < 0.05. The p-value correction method used was the Benjamini−Hochberg (BH) method.

Table S1: Results of GO and KEGG Enrichment Analysis for MRDEGs

ONTOLOGY	ID	Description	GeneRatio	BgRatio	pvalue	p.adjust	qvalue
BP	GO : 0010506	regulation of autophagy	7/44	336/18800	1.1878E-05	0.01351746	0.01064042
BP	GO : 0000422	autophagy of mitochondrion	4/44	88/18800	5.2733E-05	0.01767715	0.01391477
BP	GO : 0061726	mitochondrion disassembly	4/44	88/18800	5.2733E-05	0.01767715	0.01391477

BP	GO:0002431	Fc receptor mediated stimulatory signaling pathway	3/44	33/18800	6.2134E-05	0.01767715	0.01391477
BP	GO:0031669	cellular response to nutrient levels	5/44	213/18800	0.00013502	0.02945437	0.02318534
BP	GO:1903008	organelle disassembly	4/44	121/18800	0.00018164	0.02945437	0.02318534
BP	GO:0045542	positive regulation of cholesterol biosynthetic process	2/44	10/18800	0.00023805	0.02945437	0.02318534
BP	GO:0060368	regulation of Fc receptor mediated stimulatory signaling pathway	2/44	10/18800	0.00023805	0.02945437	0.02318534
BP	GO:0106120	positive regulation of sterol biosynthetic process	2/44	10/18800	0.00023805	0.02945437	0.02318534
BP	GO:0031668	cellular response to extracellular stimulus	5/44	245/18800	0.00025883	0.02945437	0.02318534
BP	GO:0045017	glycerolipid biosynthetic process	5/44	253/18800	0.00030015	0.03105231	0.02444317
BP	GO:0090205	positive regulation of cholesterol metabolic process	2/44	12/18800	0.0003481	0.03301141	0.0259853
BP	GO:0043484	regulation of RNA splicing	4/44	154/18800	0.00045553	0.03987652	0.03138925
BP	GO:0016241	regulation of macroautophagy	4/44	158/18800	0.00050182	0.03992	0.03142347

BP	GO：0009267	cellular response to starvation	4/44	160/18800	0.00052619	0.03992	0.03142347
BP	GO：0031667	response to nutrient levels	6/44	446/18800	0.00056684	0.04031621	0.03173536
BP	GO：0022411	cellular component disassembly	6/44	460/18800	0.0006666	0.04378803	0.03446824
BP	GO：0016236	macroautophagy	5/44	306/18800	0.00071384	0.04378803	0.03446824
BP	GO：0071496	cellular response to external stimulus	5/44	309/18800	0.00074589	0.04378803	0.03446824
BP	GO：0009991	response to extracellular stimulus	6/44	479/18800	0.00082327	0.04378803	0.03446824
BP	GO：0006839	mitochondrial transport	4/44	182/18800	0.00085259	0.04378803	0.03446824
BP	GO：0000377	RNA splicing， via transesterification reactions with bulged adenosine as nucleophile	5/44	321/18800	0.000885	0.04378803	0.03446824
BP	GO：0000398	mRNA splicing， via spliceosome	5/44	321/18800	0.000885	0.04378803	0.03446824
BP	GO：0000375	RNA splicing， via transesterification reactions	5/44	325/18800	0.00093541	0.04435401	0.03491376
BP	GO：0042594	response to starvation	4/44	196/18800	0.00112257	0.04979609	0.03919755

BP	GO：0051591	response to cAMP	3/44	89/18800	0.00118042	0.04979609	0.03919755
BP	GO：0050765	negative regulation of phagocytosis	2/44	22/18800	0.00120036	0.04979609	0.03919755
BP	GO：0010893	positive regulation of steroid biosynthetic process	2/44	23/18800	0.00131273	0.04979609	0.03919755
BP	GO：0045540	regulation of cholesterol biosynthetic process	2/44	23/18800	0.00131273	0.04979609	0.03919755
BP	GO：0106118	regulation of sterol biosynthetic process	2/44	23/18800	0.00131273	0.04979609	0.03919755
CC	GO：0005769	early endosome	6/44	389/19594	0.00021977	0.03403	0.02570493
CC	GO：0030904	retromer complex	2/44	12/19594	0.00032065	0.03403	0.02570493
CC	GO：0005774	vacuolar membrane	6/44	449/19594	0.00047264	0.03403	0.02570493

GO，Gene Ontology；BP，Biological Process；CC，Cellular Component。

Table S2 Results of GSEA for MI Combined Datasets

ID	Set Size	Enrichment Score	NES	p value	p adjust	q value
THEILGAARD_NEUTROPHIL_AT_SKIN_WOUND_DN	176	0.455988	2.072884	0.002415	0.038404	0.032332
ALTEMEIER_RESPONSE_TO_LPS_WITH_MECHANICAL_VENTILATION	92	0.558472	2.302181	0.002342	0.038404	0.032332
BLANCO_MELO_BETA_INTERFERON_	144	0.483112	2.134271	0.002404	0.038404	0.032332

TREATED_BRONCHIAL_EPITHELIAL_CELLS_DN						
WP_INTERACTIONS_OF_NATURAL_KILLER_CELLS_IN_PANCREATIC_CANCER	17	-0.75314	-2.11871	0.001923	0.038404	0.032332
WP_TGFBETA_RECEPTOR_SIGNALING	48	-0.58001	-2.09596	0.001852	0.038404	0.032332
PID_IL12_STAT4_PATHWAY	29	-0.65161	-2.07735	0.001953	0.038404	0.032332
WP_TGFBETA_RECEPTOR_SIGNALING_IN_SKELETAL_DYSPLASIAS	50	-0.57075	-2.0738	0.001842	0.038404	0.032332
CURSONS_NATURAL_KILLER_CELLS	13	-0.90134	-2.32434	0.001876	0.038404	0.032332
ZHENG_IL22_SIGNALING_UP	42	0.583161	2.058976	0.00211	0.038404	0.032332
NABA_ECM_AFFILIATED	112	0.461281	1.981146	0.002294	0.038404	0.032332
REACTOME_TP53_REGULATES_TRANSCRIPTION_OF_CELL_DEATH_GENES	37	0.573976	1.976146	0.002088	0.038404	0.032332
BILD_CTNNB1_ONCOGENIC_SIGNATURE	60	-0.52793	-1.97085	0.001828	0.038404	0.032332
BIOCARTA_NO2IL12_PATHWAY	14	-0.74401	-1.96409	0.001866	0.038404	0.032332
REACTOME_ACTIVATION_OF_IRF3_IRF7_MEDIATED_BY_TBK1_IKK_EPSILON	14	0.754576	1.957491	0.002146	0.038404	0.032332
MILI_PSEUDOPODIA_HAPTOTAXIS_UP	361	-0.41218	-1.9569	0.001555	0.038404	0.032332
GROSS_HYPOXIA_VIA_ELK3_ONLY_UP	29	0.5911	1.921756	0.002041	0.038404	0.032332
REACTOME_DECTIN_2_FAMILY	15	0.712643	1.903255	0.002105	0.038404	0.032332

WP_IL4_SIGNALING_PATHWAY	48	0.519875	1.893474	0.002165	0.038404	0.032332
BLANCO_MELO_COVID19_BRONCHIAL_EPITHELIAL_CELLS_SARS_COV_2_INFECTION_UP	108	0.433863	1.859034	0.002273	0.038404	0.032332
MATSUDA_NATURAL_KILLER_DIFFERENTIATION	370	-0.3897	-1.85355	0.001555	0.038404	0.032332
WP_DEVELOPMENT_AND_HETEROGENEITY_OF_THE_ILC_FAMILY	30	-0.57531	-1.85321	0.001927	0.038404	0.032332

MI, Myocardial Infarction；GSEA, Gene Set Enrichment Analysis。

Table S3 Results of GSEA for T2D Combined Datasets

ID	Set Size	Enrichment Score	NES	p value	p adjust	q value
BLANCO_MELO_COVID19_BRONCHIAL_EPITHELIAL_CELLS_SARS_COV_2_INFECTION_UP	99	0.710464	2.843926	0.002024	0.016034	0.011101
WP_OVERVIEW_OF_PROINFLAMMATORY_AND_PROFIBROTIC_MEDIATORS	45	0.782927	2.676548	0.002101	0.016034	0.011101
ALTEMEIER_RESPONSE_TO_LPS_WITH_MECHANICAL_VENTILATION	88	0.644517	2.50158	0.002049	0.016034	0.011101
BLANCO_MELO_BETA_INTERFERON_TREATED_BRONCHIAL_EPITHELIAL_CELLS_UP	245	0.528438	2.367904	0.002331	0.016034	0.011101
SEKI_INFLAMMATORY_RESPONSE_LPS_UP	56	0.65544	2.354799	0.002037	0.016034	0.011101
TURASHVILI_BREAST_DUCTAL_CARCINOMA_VS_DUCTAL_NORMAL_DN	125	0.555793	2.305388	0.002045	0.016034	0.011101
HOLLERN_EMT_BREAST_TUMOR_DN	78	0.602648	2.29996	0.002049	0.016034	0.011101
MAHAJAN_RESPONSE_TO_IL1	65	0.612508	2.247946	0.002	0.016034	0.011101

A_UP						
BLAN-CO_MELO_BRONCHIAL_EPITHELI-AL_CELLS_INFLUENZA_A_DEL_NS1_INFECTION_UP	391	0.473181	2.232237	0.002315	0.016034	0.011101
REAC-TOME_INTERLEUKIN_10_SIGNALING	32	0.6974	2.228034	0.002096	0.016034	0.011101
GROSS_HYPOXIA_VIA_ELK3_DN	119	0.536786	2.215293	0.002053	0.016034	0.011101
FOROUTAN_PRODRANK_TGFB_EMT_UP	175	0.510098	2.195534	0.002137	0.016034	0.011101
COU-LOUARN_TEMPORAL_TGFB1_SIGNATURE_UP	86	0.565495	2.194223	0.002012	0.016034	0.011101
CHEN_LVAD_SUPPORT_OF_FAILING_HEART_UP	86	0.563493	2.186455	0.002012	0.016034	0.011101
REAC-TOME_INTEGRATION_OF_ENERGY_METABOLISM	84	-0.57901	-2.16267	0.001946	0.016034	0.011101
ZHOU_INFLAMMATORY_RESPONSE_LIVE_UP	246	0.476986	2.141172	0.00232	0.016034	0.011101
FOROUTAN_TGFB_EMT_UP	182	0.489312	2.12796	0.002079	0.016034	0.011101
BIOCAR-TA_IL1R_PATHWAY	27	0.692635	2.123281	0.002105	0.016034	0.011101
WP_CYTOKINES_AND_INFLAMMATO-RY_RESPONSE	15	0.783656	2.083893	0.002083	0.016034	0.011101
RYAN_MANTLE_CELL_LYMPHO-MA_NOTCH_DIRECT_UP	98	0.521226	2.073126	0.002075	0.016034	0.011101

T2D，Type 2 Diabetes；GSEA，Gene Set Enrichment Analysis。

Table S4 Results of GSVA for MI Combined Datasets

ID	logFC	AveExpr	t	P.Value	adj.P.Val	B
HALLMARK_HEME_METABOLISM	-0.14610248	-0.004884693	-2.098997275	0.038135135	0.563709987	-3.659766174
HALLMARK_MYOGENESIS	-0.127628911	-0.019639614	-2.073676261	0.04047493	0.563709987	-3.699330038
HALLMARK_APICAL_JUNCTION	-0.107771301	-0.009669004	-2.044752492	0.043297625	0.563709987	-3.743990239
HALLMARK_WNT_BETA_CATENIN_SIGNALING	-0.114349101	0.001089897	-2.027149253	0.045096799	0.563709987	-3.770891657

MI，Myocardial Infarction；GSVA，Gene Set Variation Analysis。

Table S5 Results of GSVA for T2D Combined Datasets

ID	logFC	AveExpr	t	P.Value	adj.P.Val	B
HALLMARK_PANCREAS_BETA_CELLS	-0.488961468	0.034172136	-3.849606395	0.000247756	0.012387801	0.30023626
HALLMARK_MYC_TARGETS_V1	0.341744354	-0.019057608	3.426094677	0.000998772	0.019594141	-0.974483486
HALLMARK_TNFA_SIGNALING_VIA_NFKB	0.353361792	0.009482847	3.280317105	0.00157754	0.019594141	-1.388809477
HALLMARK_E2F_TARGETS	0.316444565	-0.024033495	3.230679877	0.00183805	0.019594141	-1.526859621
HALLMARK_ALLOGRAFT_REJECTION	0.31815627	-0.01826364	3.209773203	0.001959414	0.019594141	-1.584535496
HALLMARK_MYC_TARGETS_V2	0.360042597	-0.045221604	3.112671874	0.002627956	0.020984813	-1.848710953

HALLMARK_ANDROGEN_RESPONSE	0.298690688	-0.012131133	3.075296827	0.002937874	0.020984813	-1.948748876
HALLMARK_G2M_CHECKPOINT	0.238576468	-0.034174638	2.388766195	0.019433167	0.121457296	-3.612390105
HALLMARK_IL6_JAK_STAT3_SIGNALING	0.242691013	-0.015104973	2.312117326	0.023531404	0.130730022	-3.776403787
HALLMARK_PI3K_AKT_MTOR_SIGNALING	0.223447524	0.009889399	2.249505759	0.027428919	0.137144594	-3.906955701
HALLMARK_MYOGENESIS	-0.202745666	-0.01795072	-2.087284323	0.040276365	0.183074386	-4.23060981

T2D, Type 2 Diabetes；GSVA, Gene Set Variation Analysis。

Table S6: mRNA-TF.

mRNA	TF
ATG14	CTCF
ATG14	FOXA1
ATG14	FOXA2
ATG14	JUND
ATG14	MAX
ATG14	MYC
ATG14	RAD21
ATG14	STAT3
ATG14	USF1
ATG14	USF2
BIRC2	CREB1
BIRC2	RELA
CSK	NRF1
FBXO7	ELF1
FBXO7	NRF1

SNX6	CEBPB
SNX6	CTCF
SNX6	ERG
SNX6	FOS
SNX6	FOSL2
SNX6	FOXA1
SNX6	GATA1
SNX6	GATA2
SNX6	JUN
SNX6	JUND
SNX6	MED1
SNX6	NRF1
SNX6	SPI1
SNX6	TEAD4
TFEB	JUN
TFEB	JUND
TFEB	MAX
TFEB	MYC
TFEB	RAD21
TFEB	RELA
TFEB	RUNX1
TFEB	SMARCA4
TFEB	SMC3
TFEB	SPI1
TFEB	STAG1
TFEB	TEAD4
TFEB	ZNF143
TFEB	CEBPB
TFEB	CTCF
TFEB	ELF1

TFEB	ERG
TFEB	ESRRA
TFEB	FOSL2
YES1	CEBPB
YES1	SPI1
